# Structural insights into the human NuA4/TIP60 acetyltransferase and chromatin remodeling complex

**DOI:** 10.1126/science.adl5816

**Published:** 2024-08-23

**Authors:** Zhenlin Yang, Amel Mameri, Claudia Cattoglio, Catherine Lachance, Alfredo Jose Florez Ariza, Jie Luo, Jonathan Humbert, Deepthi Sudarshan, Arul Banerjea, Maxime Galloy, Amélie Fradet-Turcotte, Jean-Philippe Lambert, Jeff A. Ranish, Jacques Côté, Eva Nogales

**Affiliations:** 1 California Institute for Quantitative Biosciences (QB3), University of California, Berkeley, Berkeley, CA, USA.; 2 Howard Hughes Medical Institute, University of California, Berkeley, Berkeley, CA, USA.; 3 St-Patrick Research Group in Basic Oncology, Oncology Division of the CHU de Québec-Université Laval Research Center, Laval University Cancer Research Center, Quebec City, QC, Canada.; 4 Department of Molecular and Cell Biology, University of California, Berkeley, CA, USA.; 5 Biophysics Graduate Group, University of California, Berkeley, CA, USA.; 6 Institute for Systems Biology, Seattle, WA, USA.; 7 Endocrinology Division of the CHU de Québec-Université Laval Research Center, Laval University Cancer Research Center, Quebec City, QC, Canada.; 8 Molecular Biophysics and Integrative Bioimaging Division, Lawrence Berkeley National Laboratory, Berkeley, CA, USA.

## Abstract

**INTRODUCTION:**

Covalent and noncovalent modifications of chromatin alter its structure and stability and are critical for the regulation of gene expression. The human Tat-interactive protein, 60 kDa (TIP60) complex, a fusion of the yeast nucleosome acetyltransferase of histone H4 (NuA4) and switch/sucrose nonfermentable related 1 (SWR1) complexes, is involved in both histone acetylation and H2A.Z-H2B histone exchange and is essential for cell viability, playing a role, among other cellular processes, in gene regulation and DNA double-strand break repair. Mutations in TIP60 have been linked to various disorders, including cancer and Alzheimer’s disease. However, we currently lack an understanding of its assembly and the mechanisms behind its dual functionality. TIP60 interacts with transcription factors and can be recruited to specific loci through its largest subunit, transformation/transcription domain-associated protein (TRRAP), which is shared with the Spt-Ada-Gcn5 acetyltransferase (SAGA) complex. How TRRAP is partitioned between these two large complexes and its specific role in TIP60 remain to be explored.

**RATIONALE:**

The human TIP60 complex has a molecular mass of >1.5 MDa and contains 17 or more distinct subunits organized in several functional modules that recognize and modify chromatin. In our study, we used biochemical assays, cryo–electron microscopy (cryo-EM), and cross-linking mass spectrometry (CX-MS) to investigate the coordination of TIP60 activities, elucidate its subunit organization, and shed light on its engagement with the nucleosome. Guided by our structural data, we conducted functional genomic assays in cells to reveal the role of the TRRAP module, the coordination of acetylation and histone exchange, and the regulation of gene expression across the genome.

**RESULTS:**

We show that the large E1A binding protein P400 (EP400), which contains regions corresponding to the Swr1 subunit of the yeast SWR1 complex and to the Esa1-associated factor 1 (Eaf1) subunit from the NuA4 complex, plays a scaffolding role within TIP60, directly partitioning into or interacting with its five functional modules: trimer independent of NuA4 involved in transcription interactions with nucleosomes (TINTIN), histone acetyltransferase (HAT), TRRAP, actin-related protein (ARP), and BASE modules. EP400 contains a distinct, extended region following the motor domain, including a segment that integrates within the ARP module and tethers the HAT module, and segments that bind to TRRAP. The HAT module is flexibly attached, and we found that although the acetyltransferase activity of TIP60 stimulates H2A.Z-H2B exchange, the reverse is not true. The ARP module, which is centrally located and coordinates the arrangement of the rest of the complex, presents a new position with respect to the hexameric AAA–adenosine triphosphatase compared with its counterparts, the SRCAP and SWR1 remodeling complexes. Upon binding to the nucleosome, the ARP module undergoes a conformational change reminiscent of but likely distinct from that observed for the SRCAP complex. We show that TRRAP engages with the Swi3, Ada2, N-Cor, and TFIIIB (SANT) domain and hydrophobic domain (HD) of the EP400 subunit through regions that are also used for interaction with the SAGA complex, thereby preventing the formation of hybrid complexes. Deletion of the SANT and HD domains of EP400 leads to the detachment of the TRRAP module, the relocalization of TIP60 in the genome, and a redistribution of H2A.Z and its acetylation that affects gene expression patterns.

**CONCLUSION:**

Our studies define the structural organization of TIP60, shed light on the coordination of H2A.Z-H2B exchange and histone acetylation activities, and demonstrate the functional importance of the TRRAP module in defining regions of activity in the genome. These findings contribute to our molecular understanding of TIP60 assembly and structure and are relevant to a variety of human diseases. ■

## Abstract (summary)

The human nucleosome acetyltransferase of histone H4 (NuA4)/Tat-interactive protein, 60 kilodalton (TIP60) coactivator complex, a fusion of the yeast switch/sucrose nonfermentable related 1 (SWR1) and NuA4 complexes, both incorporates the histone variant H2A.Z into nucleosomes and acetylates histones H4, H2A, and H2A.Z to regulate gene expression and maintain genome stability. Our cryo–electron microscopy studies show that, within the NuA4/TIP60 complex, the E1A binding protein P400 (EP400) subunit serves as a scaffold holding the different functional modules in specific positions, creating a distinct arrangement of the actin-related protein (ARP) module. EP400 interacts with the transformation/transcription domain-associated protein (TRRAP) subunit by using a footprint that overlaps with that of the Spt-Ada-Gcn5 acetyltransferase (SAGA) complex, preventing the formation of a hybrid complex. Loss of the TRRAP subunit leads to mislocalization of NuA4/TIP60, resulting in the redistribution of H2A.Z and its acetylation across the genome, emphasizing the dual functionality of NuA4/TIP60 as a single macromolecular assembly.

## Graphical Abstract

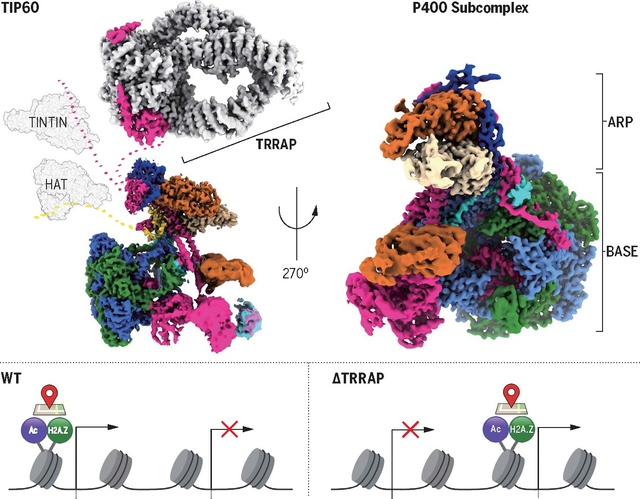

**Architecture of the TIP60 complex and its role in gene expression through histone acetylation and H2A.Z-H2B exchange.** Within the human TIP60 complex, modules for histone acetylation, H2A to H2A.Z exchange, and localization to active enhancers and gene promoters extend from a central scaffold that coordinates these distinct activities. The complex contains five functional modules: TRRAP, TINTIN, HAT, ARP, and BASE (top). The ARP module adopts a new orientation relative to the BASE module (top right), likely required to incorporate dual functionalities. TIP60 is recruited to specific genome loci, where it coordinates acetylation and deposition of H2A.Z-H2B dimers to promote gene expression (bottom left). In the absence of TRRAP (DTRRAP), TIP60 mislocalizes, leading to altered patterns of chromatin states and gene expression (bottom right). WT, wild type; ac, acetyl group.

Alteration of the chromatin state to change the accessibility of DNA regions is carried out by protein complexes that either covalently modify histones or use adenosine triphosphate (ATP) hydrolysis to change the position and/or composition of nucleosomes, thus allowing eukaryotic cells to regulate replication, transcription, and DNA repair. Histone posttranslational modifications (PTMs), such as acetylation, can alter the interaction of histone tails with DNA and the acidic patch on nucleosomes and affect the availability of the tails for further modifications. Complex epigenetic cross-talk can be established by the recruitment of proteins containing reader modules for specific histone PTMs, including ATP-dependent chromatin remodelers and other histone modifiers. As an example, the essential yeast nucleosome acetyltransferase of histone H4 (NuA4) complex is recruited by transcription factors to acetylate histones H4 and H2A at gene promoters, allowing binding of other factors, recruitment of RNA polymerase II, and, in some contexts, stimulating the switch/sucrose nonfermentable related 1 (SWR1) chromatin remodeler. In turn, SWR1 uses ATP hydrolysis to replace canonical histone H2A with the H2A.Z variant in promoter-proximal nucleosomes (1, 2). In humans, the large NuA4 and SWR1 complexes have “merged” into a single supramolecular entity, the NuA4/TIP60 complex (referred to from here on as TIP60).

The human TIP60 complex has a molecular mass of >1.5 MDa and contains >17 distinct subunits, depending on cell type, which are distributed in several functional modules (3–8) (fig. S1A). TIP60 is essential for cell viability, as it participates in critical cellular processes, including gene regulation and DNA double-strand break repair. It also has critical functions guiding cell fate, being required for stem cell maintenance and renewal, regulating the cell cycle, differentiation, apoptosis, and autophagy, with increasing reports of its function in cell metabolism (6, 9–13). Furthermore, TIP60 plays critical roles in brain development and pathologies (9, 10, 14, 15), and mutations in this complex have been linked to neurodevelopmental disorders (16–19) as well as cancer (20–23). TIP60 can modify nucleosomes through covalent and noncovalent mechanisms by using its histone acetyltransferase (HAT) module and its E1A binding protein P400 (EP400) chromatin remodeler subunit, respectively. It can also recognize chromatin states through several reader domains for different histone marks (4, 24–31) (fig. S1A). The EP400 subunit contains regions corresponding to the Swr1 subunit of the yeast SWR1 complex and to the Esa1-associated factor 1 (Eaf1) subunit from the NuA4 complex (32). It harbors a switch 2/sucrose nonfermentable 2 (SWI2/SNF2) family adenosine triphosphatase (ATPase) motor that belongs to the inositol auxotrophy 80 (INO80) family of chromatin remodelers (25, 33). Whereas TIP60 is an apparent merge of yeast NuA4 and SWR1 complexes, another human complex, Snf2 related CREBBP activator protein (SRCAP), corresponds more closely to SWR1 (34) (fig. S1B). Depletion of EP400 or SRCAP has been shown to affect H2A.Z levels at specific genomic loci in vivo (15, 31, 35–37). Specific subunits shown to be essential for this histone exchange by the SWR1 complex, actin-related protein 6 (Arp6) and Zinc finger HIT-type containing 1 (ZNHIT1), are present within SRCAP but absent from TIP60 (fig. S1, A and B), suggesting distinct mechanisms for remodeling chromatin (38, 39). H2A.Z contributes to different gene regulation outcomes depending on the gene context and its modification status (2, 40, 41). Of note, H2A.Z acetylation is strictly dependent on NuA4 in yeast and TIP60 in mammals (9, 42–45). Of clear relevance for potential therapeutic strategies, a recent report showed that loss of EP400 is synthetic lethal with perturbation of the SWI/SNF remodeler in cancer cell lines (46), a link supported by CRISPR screens and cancer patient data. This study of cells lacking a functional SWI/SNF remodeler found that EP400 has a compensatory role, being required to reestablish chromatin accessibility at most promoters (46–48).

The structures of the yeast NuA4 complex (49–54) and members of the INO80 family of chromatin remodelers (33, 55–60) have been extensively studied, providing insights into their architecture and function. However, the structural organization of the human TIP60 complex, a hybrid complex comprising elements from both SWR1 and NuA4, remains unknown.

## Purified, native TIP60 complex coordinates H2A.Z exchange and acetyltransferase activities within a single complex

**Fig. 1.**
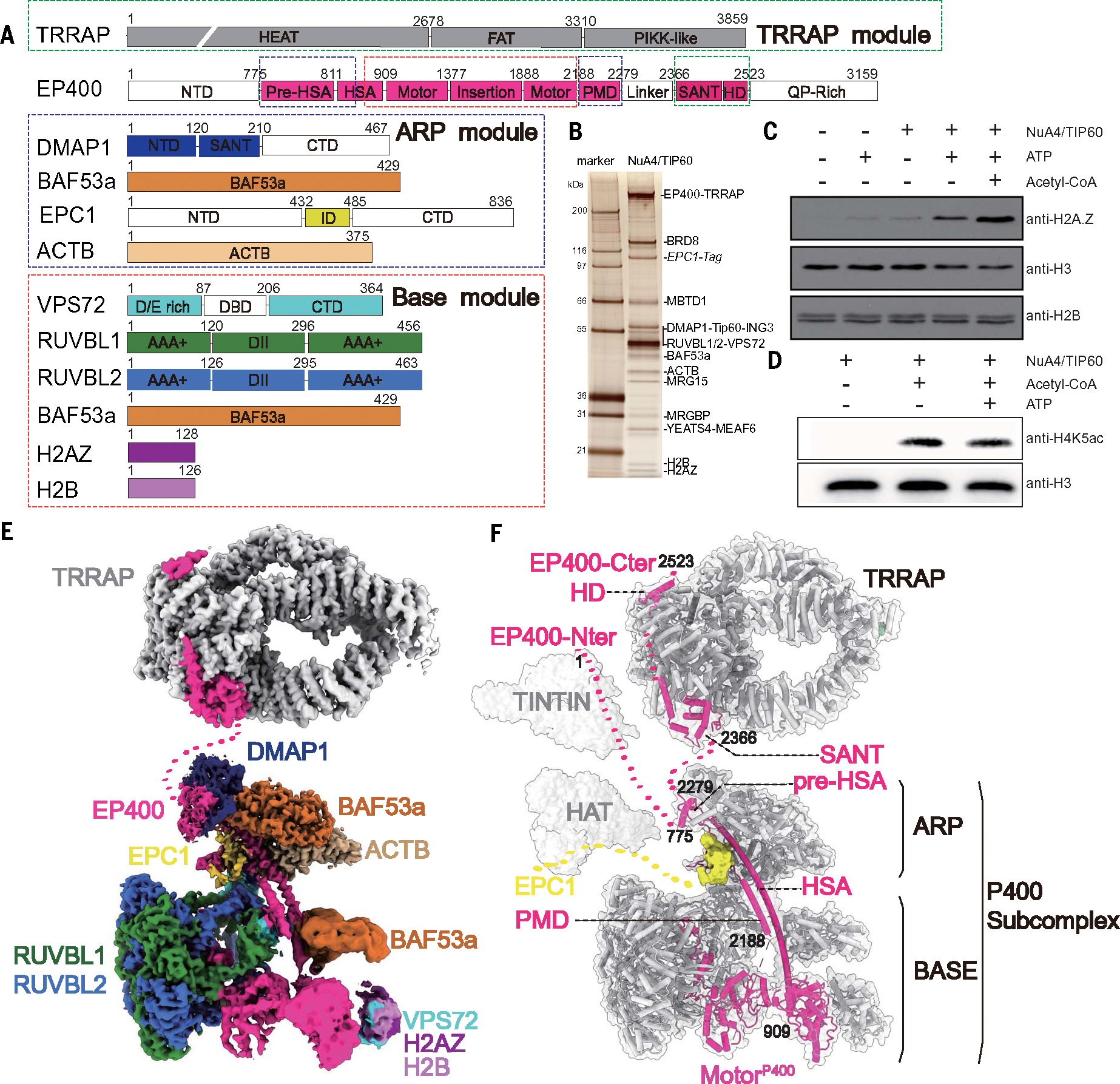
Overall structure of the human NuA4/TIP60 complex. (**A**) Domain architecture of individual subunits within the TIP60 complex that were visualized in this study, with regions modeled shown in color and unmodeled regions shown in white. Residue numbers at boundary regions are indicated. The visualized subunits are associated into three main modules, indicated by the dashed boxes. The TRRAP module comprises the TRRAP subunit and the SANT and HD domains of EP400. The P400 subcomplex consists of two modules: ARP and BASE. The ARP module includes EPC1, DMAP1, BAF53a, and ACTB as well as the pre-HSA, HSA, and PMD domains of P400. The BASE module consists of three copies each of RUVBL1 and RUVBL2 as well as one each of VPS72, BAF53a, H2AZ, H2B, and the Insertion and Motor domains of EP400. The same color code for the subunits is used throughout the figures. (**B**) Silver-stained gel of native TIP60 complex purified from K562 cells expressing EPC1–3xFlag-2xStrep (tandem affinity purification from nuclear extracts). Subunits are identified on the right. (**C**) Histone exchange assay carried out with purified native TIP60 by using recombinant dinucleosomes on beads and added H2A.Z-H2B dimers. Efficient H2A.Z incorporation was detected by immunoblotting in the presence of the complex, ATP, and acetyl-CoA when indicated. H3 and H2B signals served as controls to show the amount of nucleosomes present in the reactions. (**D**) The HAT assay was performed on nucleosomes BY using native TIP60 in the presence of Acetyl-CoA and ATP when indicated. HAT activity was assessed with a H4K5ac antibody, with the H3 signal serving as loading control. (**E**) Front view of the cryo-EM map of endogenous TIP60, with the subunits colored as depicted in (A). Structures of the P400 subcomplex (a composite map of the ARP module, BASE module, and VPS72-H2AZ-H2B) and the TRRAP module. (**F**) Localization of the different regions of EP400 within the TIP60 complex. EP400 and EPC1 are depicted in hot pink and yellow, respectively, whereas the remaining subunits are shown in gray. The TINTIN and HAT modules are flexibly attached to the rest of the complex and were not visible in our reconstruction (except for part of EPC1 that connects the HAT module to the rest of the complex). Light gray cartoons are used as a schematic indication of each module and provide an estimated localization rather than a precise structural depiction (same for Fig. 2A).

With the goal of structural and functional characterization of the TIP60 complex, we purified the native, endogenous complex from K562 cells that had been genome engineered to produce a tagged version of the enhancer of polycomb homolog 1 (EPC1) subunit specific to TIP60 (28). After tandem-affinity purification from nuclear extracts, biochemical and mass spectrometry (MS) analysis showed the presence of 17 distinct subunits (Fig. 1B). We used the purified fractions to test the activity of the native complex to replace H2A with H2A.Z in nucleosomes. Swapping assays on recombinant dinucleosomes showed incorporation of H2A.Z in an ATP-dependent fashion (Fig. 1C and fig. S1E). This activity was similar to that obtained with the native SRCAP complex purified in parallel (fig. S1, C to F). Thus, our results indicate that the absence of Arp6 and ZNHIT1 (Vps71 in yeast SWR1) in TIP60 (fig. S1, A and B) does not affect its ability to incorporate H2A.Z, suggesting a mechanism of remodeling distinct from that of the yeast SWR1 and human SRCAP complexes. Next, we determined whether the H2A.Z exchange activity could be stimulated by the acetyltransferase activity and observed a marked increase in H2A.Z exchange when acetyl–coenzyme A (CoA) was present in the reaction (Fig. 1C, last lane). However, the HAT activity was not affected by the H2A.Z exchange activity, as the presence of ATP and H2A.Z dimers did not affect the reaction (Fig. 1D). These results indicate that acetyltransferase activity can substantially boost H2A.Z histone exchange, although the converse is not true. Coupled with previous reports by us and others that the yeast SWR1 complex can more efficiently incorporate H2A. Z into nucleosomes preacetylated by NuA4 (61, 62), our current findings underscore the coordination between a chromatin modifier and an ATP-dependent remodeler across different species, which, in the metazoan case, reside in a single protein complex.

## Overall structure of the human TIP60 complex

Purified TIP60 was mildly crosslinked and used for cryo–electron microscopy (cryo-EM) characterization (fig. S2). Two-dimensional (2D) class averages displayed a well-defined density corresponding to less than half of the expected mass of the complex and an associated halo of blurred density indicative of large flexibility for the other half (fig. S2C). We were able to generate a density map with an overall resolution of 5.3 Å of what we refer to as the P400 subcomplex, which includes the ARP and BASE modules (Fig. 1, E and F, and fig. S2). Local refinement led to a reconstruction of the ARP and BASE modules with a resolution of 3.3 Å into which we built atomic models for EPC1, DNA methyltransferase 1–associated protein 1 (DMAP1), actin beta (ACTB), part of the large EP400 subunit, two copies of BRG1-associated factor 53a (BAF53a), and three copies of alternating RuvB-like 1 (RUVBL1) and RUVBL2 forming the heterohexameric AAA+ (ATPases associated with diverse cellular activities) ring, (Fig. 1, A, E, and F, and fig. S3, A and B). Using focused classification and refinement, we also identified a distinct density proximal to the EP400 ATP-dependent motor corresponding to the vacuolar protein sorting 72 homolog (VPS72 or YL-1) chaperone subunit bound to an H2A.Z-H2B dimer (Fig. 1, A, E, and F, and figs. S2, D and E, and S3C). Although a crystal structure has been reported for VPS72 bound to the H2AZ-H2B dimer (63, 64), this module has been absent from previous structural studies of the yeast SWR1 or human SRCAP complexes (33, 56, 57, 60, 65).

Our structural model of the endogenous TIP60 complex does not include the trimer independent of NuA4 involved in transcription interactions with nucleosomes (TINTIN) module, and only a small segment of the HAT module could be traced (EPC1), as they are flexibly tethered to the rest of the complex. The TINTIN module was expected to be flexibly attached to the rest of the complex through the EP400 N-terminal domain (NTD) (30, 49, 52). As is also the case for the yeast NuA4 complex, the HAT module is flexibly tethered to the P400 subcomplex through a previously identified region within EPC1 (49–54). AlphaFold (66, 67) predicts that region to be connected to the rest of the HAT by a long, unstructured segment of 140 amino acids (fig. S2I). We found that our reconstructions did not include density for the transformation/transcription domain-associated protein (TRRAP) either. TRRAP (Tra1 in yeast), which is the largest subunit in the complex and is involved in its recruitment by transcription factors (68), is a major feature in all structural studies of the yeast NuA4 (49–54). It is shared with the SAGA complex, and the structure of human SAGA also features prominently the cradle shape of TRRAP (69, 70). Even after extensive data processing, we could only see weak, blurred density near the ARP module of EP400 that could possibly correspond to TRRAP flexibly attached to the rest of the complex (fig. S2C). To overcome this challenge, we generated a new cell line guided by our EP400 structure and chemical cross-linking mass spectrometry (CX-MS) data (Fig. 1F and fig. S4A) in which we expressed a tagged truncation of EP400 containing just the C terminus after the ATPase domain (amino acid 2188 to 3159). We used this construct for isolation of the endogenous TRRAP module (fig. S5) and obtained a 3.4-Å map of this module that enabled us to build an atomic model (Fig. 1, A, E, and F, and figs. S3D and S6). The density accounts only for the TRRAP subunit itself and two fragments of EP400 (Fig. 1A), indicating that all other components are highly flexible relative to the TRRAP module or do not associate with the EP400 C-terminal region, as indicated by the biochemical analysis of the purified fraction (fig. S5). By combining our structural and CX-MS data, we propose an overall architecture for the human TIP60 complex (Fig. 1E; for representative regions of the maps and corresponding models for different subunits, see fig. S7).

We also reasoned that unresolved, flexibly attached density present in the endogenously purified TIP60 complex could be reducing the accuracy of our image alignments and therefore the final resolution of our structure. Thus, we decided to reconstitute the P400 subcomplex, guided by our initial model of the endogenous complex, for further analysis. The more homogenous nature of the reconstituted P400 subcomplex led to a 2.6-Å cryo-EM density that allowed us to accurately assess the structural components, especially around the hexameric AAA-ATPase ring (figs. S3E and S8). Of note, the improvement in resolution and purifications of helicase-SANT–associated (HSA) mutants allowed us to confirm the presence of a second BAF53a (in addition to the one in the ARP module) bound to the HSA rather than ACTB (figs. S3F and S8, G and H), which is in contrast with the additional ACTB subunit that has been proposed to bind to the HSA domain in the SWR1 and SRCAP complexes (60, 71).

## Subunit arrangement around the EP400 scaffold

TIP60 consists of five modules, including TINTIN, HAT, TRRAP, ARP, and BASE, each with distinct functions (Fig. 1, A and F). Within the complex, the EP400 protein functions as a major scaffold, making interactions with all the functional and structural modules (Fig. 1F), a role similar to that of the Eaf1 subunit in the yeast NuA4 complex and of the Swr1 subunit in the SWR1 complex, the two subunits of which EP400 is a merge (32, 49, 50, 53, 54, 57). As it organizes the TIP60 complex, the EP400 protein is structured into 10 consecutive domains (Fig. 1, A and F) with distinct interaction partners. Although the NTD domain of EP400 is not visible in the structure, our prior work showed that this segment is a part of the TINTIN module along with proteins MRG15/X, MRGBP, and BRD8 (30). Those previous findings are consistent with our subcomplex purification using an N-terminal EP400 truncation (before the ATPase domain, amino acid 1 to 1000) and with our CX-MS results (figs. S4A and S5). Following the unresolved N-terminal domain of EP400 and preceding its motor domain, the pre-HSA region and a portion of HSA interact with DMAP1, ACTB, and one BAF53a subunit, collectively forming the ARP module (Fig. 1E–F).

The ATP-dependent motor and the insertion domain of EP400 together with the hexameric AAA-ATPase constitute most of the BASE module of the TIP60 complex below the ARP module (Fig. 1, E and F). In contrast to other members of the INO80 family (33, 55, 57, 58), EP400 contains a distinct, extended region following the motor domain that can be subdivided into five distinct structural domains: the postmotor domain (PMD), Linker, SANT domain, hydrophobic domain (HD), and the glutamine and proline (QP)–rich domain (Fig. 1, A and F). The PMD of EP400 folds back toward the N terminus, integrating itself into the ARP module and contributing to the surface that tethers the HAT module to the rest of the complex (Fig. 1F). Following a long, disordered linker, the SANT and HD domains of EP400 interact directly with the TRRAP module, linking it flexibly to the rest of TIP60 (Fig. 1F). The QP-rich domain, predicted to be disordered, is not observed in our structure.

The scaffolding role of EP400 agrees with our observation that isolation of the endogenous TIP60 complex using N- or C-terminal truncations of EP400 results in copurification with either the TINTIN and ARP modules or the TRRAP module, respectively (fig. S5). However, we were unable to copurify the HAT module with either the N- or C terminus of EP400 (fig. S5). This result is explained by our structure, as the HAT module is tethered to the rest of the complex by a short fragment of EPC1 that is sandwiched between the HSA and PMD of EP400, thus requiring both the N- and C-terminal halves of EP400 for binding.

Mutations in EP400 have been linked to schizophrenia in the Japanese population (72) that can now be mapped to different parts of the protein within our structure. Among them, rare missense variants are found in the HSA, motor, insertion, PMD, and SANT regions (fig. S9).

## The ARP module is a central hub for functional elements within TIP60

**Fig. 2.**
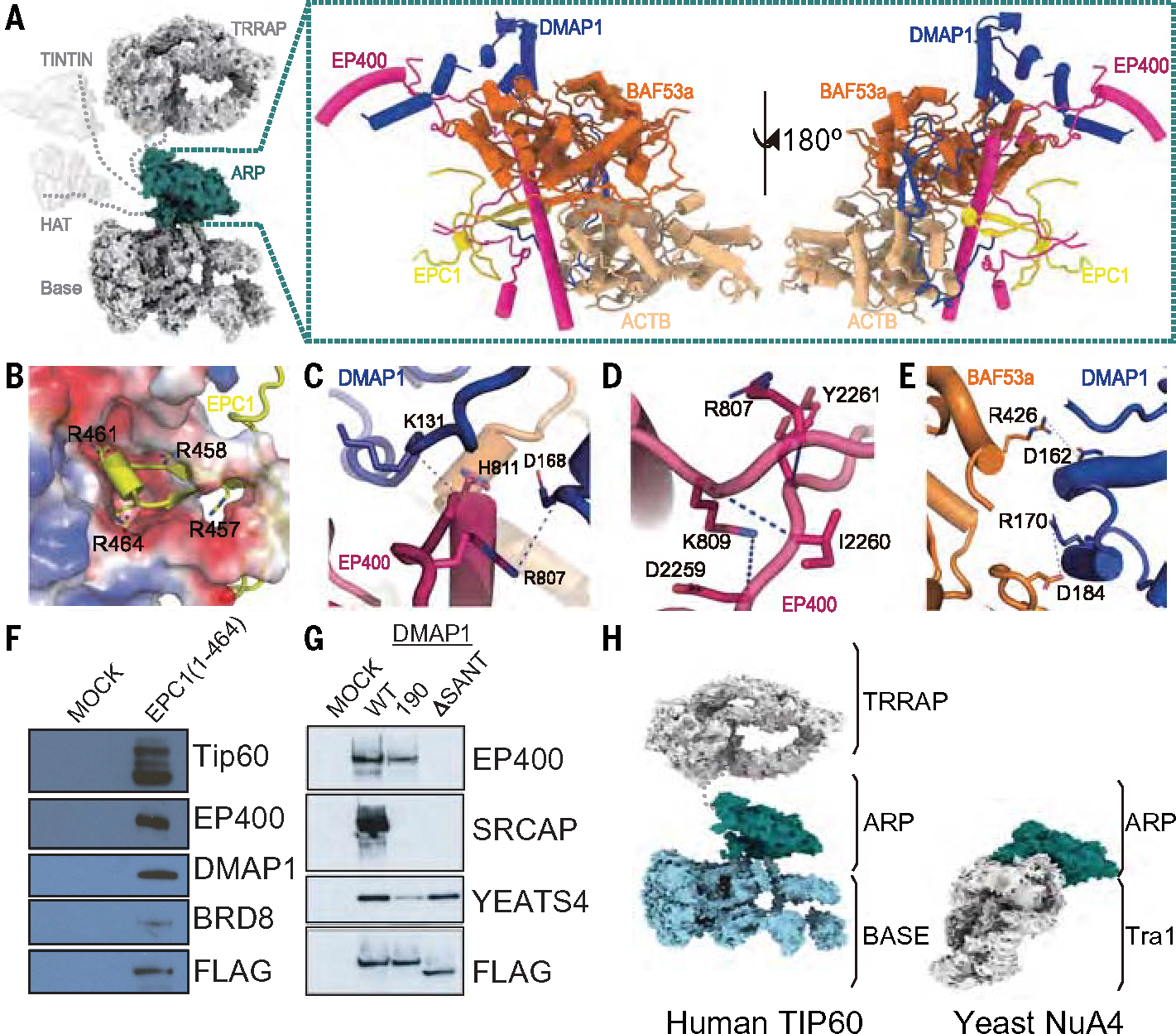
Structure of the TIP60 ARP module. (**A**) (Left) The ARP module (turquoise) is connected to all other modules within the TIP60 complex. (right) Front and back views of the ARP module are shown in ribbon diagram with subunits colored as in Fig. 1A. (**B**) A positively charged segment of EPC1 (shown as a yellow ribbon) inserts into a negatively charged cavity within the ARP module (rendered as a surface with electrostatics), tethering the HAT module to the rest of the complex. Four positively charged residues (R457, R458, R461, and R464) involved in the interaction are depicted in stick representation and labeled. (**C** to **E**) Detailed views of the interactions between (C) the pre-HSA domain of EP400 and DMAP1, (D) the pre-HSA domain and the PMD of EP400, and (E) the SANT domain of DMAP1 and BAF53a. The residues participating in the interactions are shown in stick representation and labeled. Model building was based on the cryo-EM map of ARP module (fig. S3A). (**F**) Biochemical data showing that the N-terminal amino acids up to R464 of EPC1 are sufficient to tether the HAT module to the rest of the TIP60 complex. Cells expressing EPC1(1–464)-3xFlag-2xStrep from the *AAVS1* safe harbor were used for tandem affinity purification, analyzed by immunoblotting, and compared with a mock cell line processed in parallel. See fig. S10 for silver-stained gel and MS analysis. (**G**) Biochemical data showing the critical role of the DMAP1 SANT domain and key amino acid D190 in the association of this protein with the TIP60 and SRCAP complexes. Cells expressing either WT DMAP1–3xFlag-2xStrep from the *AAVS1* safe harbor, mutant of amino acid 190 (D190A/L191A/K192A), or a mutant form lacking the SANT domain (amino acids 149 to 199; ΔSANT) were used for tandem affinity purification, analyzed by immunoblotting, and compared to a mock cell line processed in parallel. See fig. S13 for silver-stained gel and MS analysis. (**H**) Comparison of domain architecture for the human TIP60 and the yeast NuA4 complexes, aligned on their ARP domains. The position of the Tra1 subunit in NuA4 (the homolog of human TRRAP) overlaps with that of the BASE module in TIP60. Single-letter abbreviations for the amino acid residues referenced throughout this paper are as follows: R, Arg; H, His; D, Asp; K, Lys; Y, Tyr; I, Ile; L, Leu; F, Phe; E, Glu; V, Val; A, Ala; Q, Gln; P, Pro.

In the TIP60 complex, the ARP module plays a central role connecting to all other structural and functional modules (Fig. 2A), in contrast to its peripheral location in the yeast NuA4 and SWR1 complexes (49, 50, 53, 54, 57). Regions of EP400 within the module connect through flexible tethers with the TINTIN and TRRAP modules and more rigidly with the BASE module (Fig. 2A, left). The flexibly attached HAT module is tethered to the rest of the complex by a short fragment of its EPC1 subunit. Within the ARP module, the HSA of EP400 functions as a “pillar” that is surrounded by BAF53a, ACTB, DMAP1, and EPC1 (Fig. 2A, right), leaving the other half of HSA exposed to the solvent (Fig. 1, E and F). This feature contrasts with what is observed in the NuA4 complex, where the HSA is completely buried within the ARP module (49, 50, 53, 54). By contrast, in the INO80 and SRCAP complexes and also predicted for SWR1 (structural analysis has not visualized it, probably owing to flexibility), the HSA is fully exposed and can interact with nucleosomal DNA (57–60, 73, 74), contributing to the interaction of these complexes with the nucleosome. Thus, it seems that by integrating the acetylation and remodeling functions of the NuA4 and SWR1 complexes into a single entity, the HSA and ARP modules of TIP60 have evolved distinct structures and functions compared with their counterparts that possess only one of these activities.

Of the 48 amino acids of EPC1 visible in our structure (amino acid 432 to 479), a short segment (amino acid 457 to 464) interacts with a negatively charged cavity created by EP400 and DMAP1 through four positively charged residues that form salt bridges and hydrogen bonds with the cavity (Fig. 2B). This interaction was confirmed by the expression of a truncated N-terminal portion of EPC1 ending at R464, which was sufficient to anchor the HAT module to the rest of the TIP60 complex (Fig. 2F and fig. S10). It is also consistent with our EP400 truncation experiments, in which neither the N- nor C-terminal regions of EP400 in isolation could anchor the HAT module to the complex (fig. S5), emphasizing the requirement for both regions in the tethering of the HAT module. A similar tethering principle for the HAT has been observed for the yeast NuA4 complex (49, 50, 53, 54).

The pre-HSA helix of EP400 plays an important role within the core of the ARP module, where it is sandwiched between DMAP1 and the PMD of EP400 (Fig. 2A, right). Specifically, R807 and H811 in the pre-HSA interact with D168 and K131 in DMAP1, respectively (Fig. 2C), whereas R807 and K809 of the pre-HSA interact with Y2261 and with D2259 and I2260 of the PMD, respectively (Fig. 2D). Notably, the R807 residue of the pre-HSA, which participates in interactions with both DMAP1 and the PMD, has been identified as a cancer-associated mutation (R807H) (Fig. 2, C and D) (47). Additionally, D162 and R170 in the SANT domain of DMAP1 form salt bridges and hydrogen-bond interactions with R426 and D184 of BAF53a, respectively (Fig. 2E). The structural importance of these interactions was suggested when expression of DMAP1 lacking its SANT domain (amino acid 149 to 199) resulted in its dissociation from EP400 (and SRCAP) while retaining its association with the H3K27ac-reader YEATS4 subunit (Fig. 2G and fig. S11, A to B). Alanine substitutions at DMAP1 conserved residues L169, R170, and F171 detached it from both the SRCAP and TIP60 complexes, whereas L160, F161, and D162 substitutions did not (fig. S11, C and D). D190, L191, and K192 substitutions in DMAP1 selectively disrupted its interaction with SRCAP while preserving its association with TIP60 (Fig. 2G), corroborating the specific interaction of this part of the DMAP1 SANT domain with SRCAP rather than with EP400 (60).

It is notable that although the ARP modules of the human TIP60 and yeast NuA4 complexes exhibit considerable structural similarity (fig. S12), aligning the complexes on their ARP modules shows that the Tra1 module of yeast NuA4 would overlap in position with the hexameric AAA-ATPases of TIP60 (Fig. 2H) (49). Thus, TIP60 adopts a distinct architecture from that of yeast NuA4 despite sharing a similar ARP module and the TRRAP or Tra1 subunit.

## Arrangement of subunits around the hexameric AAA-ATPase ring

**Fig. 3.**
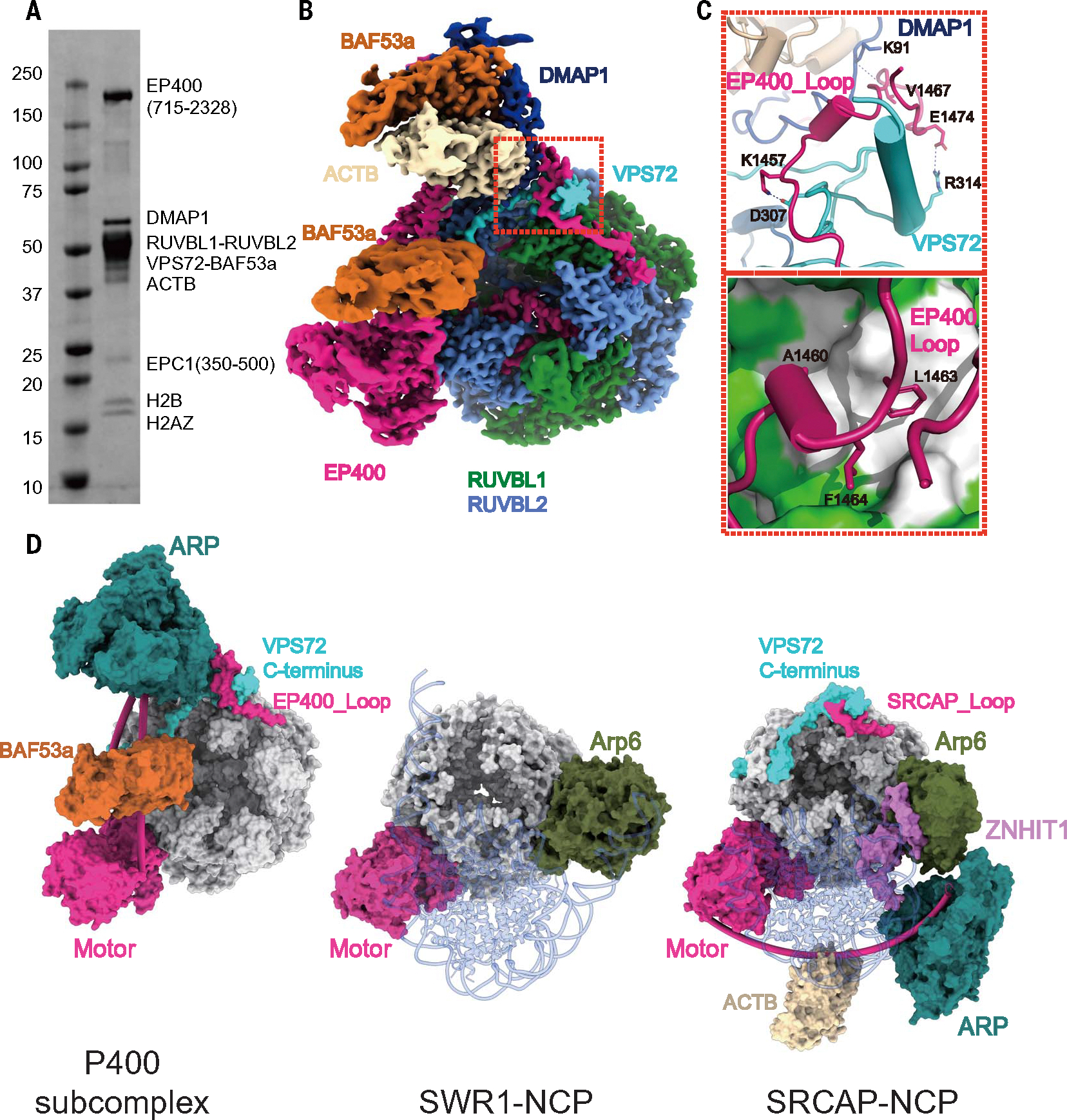
Architecture of the P400 subcomplex and comparison with other INO80 family remodelers. (**A**) SDS-PAGE gel of the reconstituted P400 subcomplex, with subunits indicated on the right. (**B**) Side view of the cryo-EM reconstruction of reconstituted P400 subcomplex (overall resolution, 2.6 Å). The connection region between the ARP and BASE modules is highlighted by the red dash box. (**C**) Zoomed-in view of the interaction region between the ARP and BASE modules. (Top) Ribbon diagram with residues involved in salt bridges and hydrogen bonds between EP400 (hot pink), VPS72 (cyan), and DMAP1 (dark blue), explicitly depicted. (Bottom) Hydrophobic interactions of EP400 (hot pink ribbon) with VPS72 and DMAP1, which are shown as a colored surface depicting hydrophobicity (green, hydrophilic; white, hydrophobic). (**D**) Side-by-side structural comparison between the P400 subcomplex described in this work (left), the SWR1-NCP complex (middle; PDB: 6GEN) and the SRCAP-NCP complex (right; PDB: 8X19). The motors of these three chromatin remodelers (hot pink) are positioned to be approximately aligned with each other [and to (B)]. Only the ARP module (turquoise), Arp6 (army green), AAA-ATPase hexamer (gray), motor and HSA domains of the remodeler (hot pink), BAF53a (brunt orange), VPS72 C terminus (cyan), ZNHIT (light purple), and ACTB (tan) are shown for clarity. The nucleosome engaged by SWR1 and SRCAP is shown for reference in gray ribbon.

The 2.6-Å cryo-EM density of the reconstituted P400 subcomplex allowed us to visualize distinct features concerning the arrangement of subunits around the hexameric AAA-ATPase ring of the TIP60 complex (Fig. 3, A to C). In the yeast SWR1 and human SRCAP complexes, the Arp6 subunit, which is not present in the TIP60 complex, interacts directly with the hexameric AAA-ATPase ring and engages the nucleosome core together with their respective motor (Fig. 3D, army green, center and right, respectively) (57, 60, 65). In addition, the TIP60 and SRCAP complexes contain an ARP module, not visible in the SWR1 structure (57), that is flexibly positioned away from the hexameric AAA-ATPase ring in the SRCAP structure (55, 57, 58) (Fig. 3D, aqua, right). By contrast, the ARP module within TIP60 is closely attached to the hexameric ring (Fig. 3, B and D, left) and in a location also completely different from that of Arp6 in the SWR1 and SRCAP complexes (Fig. 3D, center and right) (57, 60). Our structure shows that a fragment of the EP400 insertion domain (EP400_Loop) buds out of the hexameric ATPases ring and forms a series of interactions with the VPS72 C terminus and DMAP1 in the ARP module that positions it distinctly within TIP60 (Fig. 3C). The EP400_Loop is situated on the periphery of the interaction surface between the ARP and the BASE modules, whereas the VPS72 C terminus occupies a central position at the interface of the ARP and BASE modules (Fig. 3, B and C, top, and D, left). Specifically, E1474 and K1457 in the EP400_Loop form salt bridges with R314 and D307 in the VPS72 C terminus, respectively; the main chain of V1467 in the EP400_Loop forms a hydrogen bond with K91 of DMAP1 (Fig. 3C, top); and A1460, L1463, and F1464 of the EP400_Loop establish hydrophobic interactions with a cleft between DMAP1 and VPS72 C terminus (Fig. 3C, bottom). In agreement with the contacts identified in our structure, deletion of the P400_Loop substantially compromised the stability of the ARP module’s interaction with the BASE module (fig. S13, A and F). Cryo-EM analysis of this truncated complex showed that in 60% of the particles, the ARP module had dissociated from the BASE module (fig. S13F). In the remaining particles, the ARP module was retained but exhibited markedly increased flexibility (fig. S13, F and G). Of notice, mutation of the VPS72 C terminus led to a complete detachment of the ARP module from the BASE module, highlighting its essential role in mediating the engagement of the ARP module with the rest of the complex (fig. S13, B, E, and H). Moreover, the mutation of the VPS72 C terminus leads to the detachment of the VPS72-H2A.Z-H2B module, whereas this is not the case with the EP400_ Loop mutation (fig. S13, A to C), emphasizing the structural importance of the VPS72 C terminus.

## Structural differences between the human SRCAP and TIP60 complexes in the context of nucleosome engagement

Given the partial functional and compositional overlap between the TIP60 and SRCAP complexes, it is important to compare their structures and to establish their differences, especially concerning elements involved in nucleosome engagement. In contrast to what we have seen for the TIP60 complex, when not bound to the nucleosome, the SRCAP structure appears to be substantially more flexible with respect to some common components, and its structural description is missing the SRCAP_Loop, the VPS72 C terminus, and the ARP module (fig. S14, middle) (60). By contrast, in the TIP60 complex, the ARP module’s position is fixed by the P400_Loop and VPS72 C terminus, which are themselves anchored around the hexameric AAA-ATPase ring.

The recently reported structure of the SRCAP complex upon nucleosome engagement shows how Arp6 and ZNHIT1, along with the SRCAP motor and its flexibly tethered ARP module, extensively interact with the nucleosome (60), encircling it (Fig. 3D, right). In this SRCAP-nucleosome structure, ZNHIT1, which is absent in the TIP60 complex, interacts with both the H2A-H2B dimer and the DNA of the nucleosome (60). Notably, when the motor domain of EP400 is aligned with that of the SRCAP–nucleosome core particle (NCP), their ARP modules appear in completely different positions (fig. S14).

To gain information on how TIP60 engages with the nucleosome, we conducted CX-MS analysis of our P400 subcomplex in the presence of nucleosomes and adenosine-5’-(γ-thio)-triphosphate (ATPγS) (fig. S4B). As expected, we observed a number of cross-links between the nucleosome and the EP400 motor. Additionally, the ARP module, particularly the DMAP1 subunit located on the distal side from the motor in the structure of the unbound complex (Fig. 3B), exhibited multiple cross-links with the nucleosome (fig. S4B). This result suggests that the ARP module of the P400 subcomplex likely undergoes large conformational changes to bind the nucleosome, as is the case for the SRCAP complex (60). Given that the TIP60 complex shares a number of subunits with the SRCAP complex, we mapped the cross-links identified in our P400-NCP between DMAP1, BAF53a, and ACTB and the nucleosome core onto the SRCAP-NCP structure (fig. S4C). The analysis shows that 10 of the 11 cross-links between these subunits of TIP60 and the nucleosome are not compatible with the SRCAP-NCP structure. This result suggests that although the ARP module of TIP60, like that in SRCAP, may undergo conformational changes to interact with the nucleosome, it likely utilizes a distinct mode of interaction. We propose that this distinct mode of engagement may be because the ARP module also needs to serve an additional scaffolding role in the larger, bifunctional TIP60 complex (Figs. 1E and 2A).

## Incorporation of TRRAP into the TIP60 complex

**Fig. 4.**
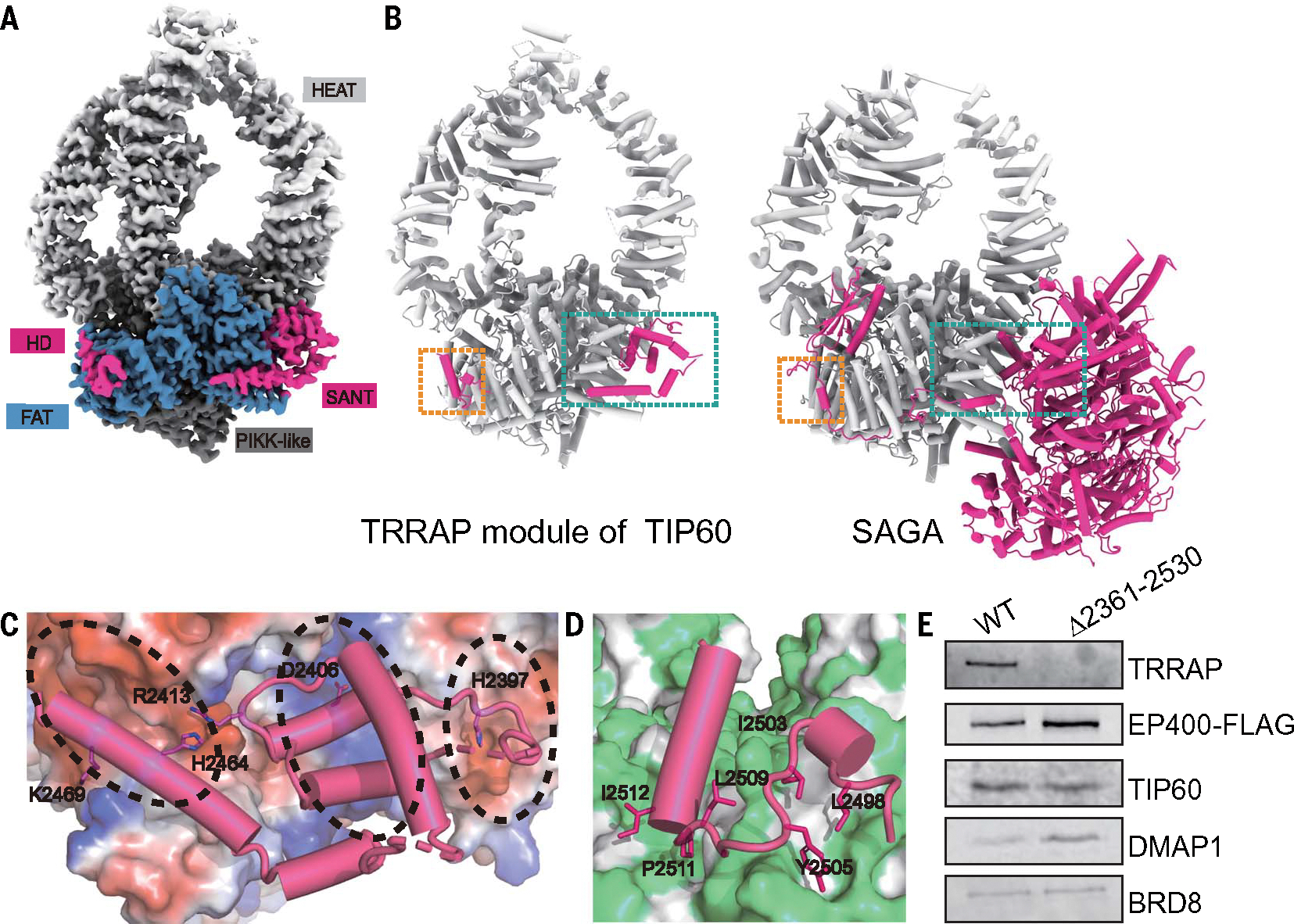
The SANT and HD domains of EP400 tether the TRRAP subunit to the rest of the complex. (**A**) Cryo-EM density map for the TRRAP module. The HEAT, FAT, and PIKK-like domains of TRRAP are depicted in light gray, steel blue, and dark gray, respectively, and EP400 segments are shown in hot pink. (**B**) Structural comparison of the TRRAP module of TIP60 (left) with human SAGA (right; PDB: 7KTR), showing TRRAP in gray and interacting elements from either complex in hot pink. The overlapping binding surfaces are highlighted by orange and aqua dashed boxes. (**C**) Detailed view of the interaction between the SANT domain of EP400 and TRRAP. TRRAP is shown as an electrostatic surface, with three distinct charged patches involved in EP400 interaction marked by dashed ovals. Key amino acids in the SANT domain participating in the interaction are shown in stick representation and labeled. (**D**) Close-up view of the interactions between the HD domain of EP400 and TRRAP. The surface representation of TRRAP shows hydrophilic regions depicted in green, and hydrophobic, in white. Residues of the HD domain of EP400 involved in hydrophobic interactions are displayed in stick representation and labeled. (**E**) Biochemical data showing the critical role of the SANT and HD domains of EP400 in the association of the TRRAP module within the rest of the TIP60 complex. Cells expressing either WT EP400–3xFlag-2xStrep from the *AAVS1* safe harbor or a mutant form lacking the SANT and HD domains (amino acids 2361 to 2530) were extracted followed by anti-Flag immunoprecipitation and Flag peptide elution and analysis by immunoblotting.

Purification of the complex by using the C-terminal segment of EP400 (amino acids 2188 to 3159) allowed us to obtain a structure of the TRRAP module in TIP60 (Figs. 1, E and F, and 4A and figs. S3D and S6). Only 137 amino acids of EP400 were resolved in our structure in addition to TRRAP itself. This fragment of EP400 corresponds to the SANT and HD domains, which interact with the focal adhesion targeting (FAT) and Huntington, elongation factor 3, PR65/A, TOR (HEAT) domains of TRRAP (Fig. 4A). Within the triangle-shaped SANT domain, residues H2397, R2413, H2464, and K2469 anchor two ends of the triangle to two acidic patches within the FAT and HEAT domains, whereas D2406, in the middle of the SANT domain, interacts with a positively charged patch of TRRAP (Fig. 4C). Sequence alignment of the SANT domains across species shows conservation of the amino acids involved in these interactions (fig. S15) (75). Of particular interest, H2397L and R2413Q have been identified as cancer-associated mutations (47). Following the SANT domain, the HD domain of EP400 interacts with the phosphatidylinositol-3 kinase-related kinase (PIKK)–like domain of TRRAP through hydrophobic interactions involving residues L2498, I2503, Y2505, L2509, P2511, and I2512. (Fig. 4D). These two binding surfaces overlap with the region of TRRAP interacting with the rest of the human SAGA complex (Fig. 4B), thus preventing the formation of hybrid assemblies between TIP60 and SAGA through a commonly incorporated TRRAP (69, 70).

The robust interactions of the SANT and HD domains with TRRAP tether it securely to the rest of the TIP60 complex (Fig. 4, A, C, and D). Guided by this structural information, we generated a cell line where the SANT and HD domains were removed from the EP400 protein, which should result in the detachment of the TRRAP module from the complex (fig. S16). Biochemical assays corroborated the TRRAP module detachment, whereas the TINTIN and HAT modules still copurified with the P400 subcomplex (Fig. 4E), as predicted by our structure. The TRRAP-interacting elements in EP400 are preceded by a region of 87 amino acids predicted to be unstructured (66, 67), thus allowing the TRRAP module a wide range of motions with respect to the rest of the complex (Fig. 1, E and F, and fig. S2C). By contrast, in the yeast NuA4, the ARP module has extensive interactions with Tra1 (49–54), rigidly positioning these two modules with respect to each other (Fig. 2H, right). In the human SAGA complex, TRRAP is also well anchored with respect to the core of that complex (Fig. 4B, right) (69, 70). Thus, the flexible linker in EP400 enables the TRRAP module to exhibit a much greater range of movement compared with that in those two complexes. Given that recruitment to specific genome loci is mediated by interaction of TRRAP with transcription factors, such as TP53, E2F, and c-Myc (76–82), the flexible tether in TIP60 would extend the reach of other functional modules within the complex that interact and modify chromatin further away from the site of recruitment, possibly allowing the complex to better adapt to the local 3D chromatin structure. TIP60 is enriched at active enhancers in stem cells, and this long reach of functional domains could potentially allow chromatin modifications and H2A.Z incorporation not only in cis configuration but also at promoters brought in close proximity to the enhancer, with possible effects on gene activation (83, 84).

## Loss of the TRRAP recruitment module leads to H2AZ redistribution in the genome

**Fig. 5.**
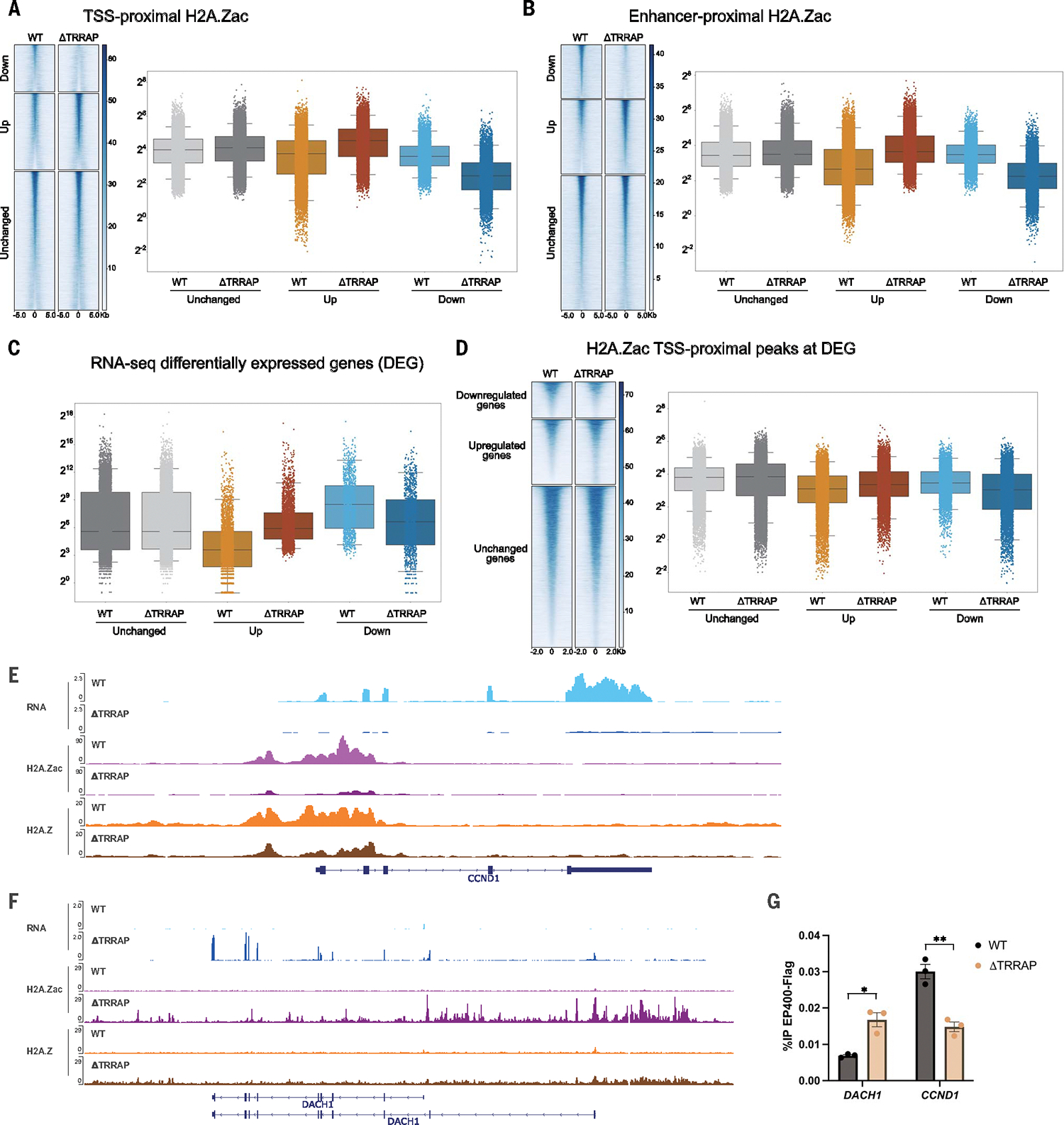
TIP60-specific function of TRRAP in vivo. (**A**) TSS-proximal H2A.Zac signal defined as the peaks located near an annotated TSS (TSS ± 2 Kb) and represented by a heatmap (left; peak center ± 5 Kb) and a box plot (right), which show regions with significant decrease (Down), significant increase (Up), or no significant change (Unchanged) in H2A.Zac signal. (**B**) Enhancer-proximal H2A.Zac signal defined as the peaks located near an annotated enhancer (enhancer ± 2 Kb) and represented by a heatmap (left; peak center ± 5 Kb) and a box plot (right), showing regions with significant decrease, significant increase, or no significant change in H2A.Zac signal. (**C**) Box plot representation of differential gene expression based on RNA-seq in the DTRRAP mutant versus the WT condition, showing normalized transcript counts on genes that are significantly down-regulated, significantly up-regulated, or with no significant change. (**D**) Heatmap and box plot representation of H2A.Zac ChIP-seq signal for peaks that are located near the TSS (± 2 Kb) of genes identified in (C). (**E**) Genome browser view of normalized read counts of RNA-seq, H2A.Zac ChIP-seq, and H2A.Z ChIP-seq over the gene *CCND1*. (**F**) Genome browser view of normalized read counts of RNA-seq, H2A.Zac ChIP-seq, and H2A.Z ChIP-seq over the gene *DACH1*. (**G**) ChIP-qPCR of FLAG-tagged EP400 showing occupancy at the promoters of *DACH1* and *CCND1* in ΔTRRAP mutant versus WT cells [error bars are standard errors from three biological replicates (*n* = 3); *P < 0.05; **P < 0.01]. ChIP-seq data are merged from two biological replicates (*n* = 2). RNA-seq data are from three biological replicates (*n* = 3). A two-sided Mann-Whitney *U* rank sum test was performed on all the box plots, and their *P* values are indicated in table S3.

To assess our structural results concerning the attachment of TRRAP to the complex and its functional importance, we created a cell line that would allow us to analyze the impact of disruptive mutations in the complex on the incorporation of H2A.Z and its acetylation in the genome as well as possible effects on gene expression. Because *EP400* is an essential gene, we produced a cell line for acute depletion of endogenous EP400 using CRISPR and the degradation tag (dTAG) approach in K562 cells to minimize indirect effects often seen with small interfering RNA (siRNA)–mediated knockdowns (validation of homozygous tagging and depletion, fig. S16, A and B). We then produced clones that expressed wild-type (WT) or mutant EP400 from the *AAVS1* safe harbour locus (fig. S16C). This system allowed us to perform functional genomic studies by fully depleting the endogenous EP400 protein and complementing it with either WT or mutant EP400 in which interaction with the TRRAP module is lost by deletion of the SANT and HD domains. We examined the distribution of H2A.Z throughout the genome as well as its acetylation, which is the best indicator of Tip60 HAT activity, as it is its main writer (9, 43, 44). The chromatin immunoprecipitation sequencing (ChIP-seq) data indicate that H2A.Z signals largely overlap with acetylated H2A.Z in the genome (nearly 80% of the peaks), supporting the combined action of Tip60 and EP400 within the complex (fig. S17,A and B). When TRRAP is lost from the complex, the number of regulatory regions that lose acetylated H2A.Z (H2A.Zac) signal is similar to the number of regions that gain signal (Fig. 5, A and B, and fig. S17, A to D). Regions that gain H2A.Z and H2A.Zac (H2A.Z/H2A.Zac) in the absence of TRRAP tend to have lower signal in WT conditions (Fig. 5, A and B, and fig. S17, B to D). Analysis of differentially expressed genes (DEGs) in the mutant cells shows that down-regulated genes tend to be highly expressed in WT conditions compared with genes that are unchanged, whereas genes that are up-regulated tend to be much less expressed in WT conditions (Fig. 5C and fig. S18C). Correlating expression and H2A.Z/H2A.Zac enrichment indicates that genes that are down-regulated in the mutant show a decrease of H2A.Z/H2A.Zac, whereas those that are up-regulated show an increase (Fig. 5D and fig. S17E). These results suggest that, in the mutant lacking TRRAP, TIP60 recruitment is lost at specific loci, which have high levels of H2A.Z/H2A.Zac and high expression in WT conditions, whereas the complex can mislocalize and act at new genomic sites that had low levels of H2A.Z/H2A.Zac and low expression in the WT but gained both in the mutant. Genes that are down-regulated and showing a drop in H2A.Z/H2A.Zac in the mutant cells are enriched in targets of transcription factors previously linked to the function of TIP60 or TRRAP as a coactivator, including MYC, E2F, HIF1a, and RelA (fig. S18D) (76, 78–82, 85, 86). An example is the gene for Cyclin D1 (*CCND1*), which shows loss of H2A.Z/H2A.Zac and transcription in the mutant cells (Fig. 5E and fig. S17G) (87). The MYC-regulated *TERT* gene (88) follows the same trends in the mutant cells compared with WT conditions (fig. S17, F and H). The *DACH1* tumor-suppressor gene (89), by contrast, shows the reverse effect, with the appearance of H2A.Z/H2A.Zac and transcription in the mutant cells (Fig. 5F).

To confirm that changes in H2A.Z/H2A. Zac and gene expression are a consequence of changes in the presence of TIP60 at those sites, we measured EP400 by ChIP–quantitative polymerase chain reaction (qPCR) in WT and mutant conditions at (i) the known TIP60 target gene *CCND1* (87), which is transcribed and has high levels of H2A.Z/H2A.Zac, and (ii) the *DACH1* gene, which is not expressed and does not show significant H2A.Z/H2A.Zac enrichment in WT conditions, although it becomes transcribed and gains the histone variant in the mutant cells. ChIP-qPCR data show the appearance of EP400 at the *DACH1* transcription start site (TSS) when TRRAP is absent from the TIP60 complex (Fig. 5G). In parallel, EP400 enrichment is decreased at the *CCND1* TSS in the same conditions (Fig. 5G), suggesting a titration effect of the TIP60 complex in the genome. These results not only confirm the structural data that identified a critical region of EP400 in anchoring the TRRAP module to the complex but also demonstrate the role of TRRAP within TIP60 in targeting the complex at specific regulatory elements in the genome to stimulate gene expression.

## Discussion

The human TIP60 coactivator complex brings together two distinctive functional roles that, in yeast, are carried out by two different complexes, SWR1 and NuA4, with its key subunit EP400 being a “fusion” of the Swr1 and Eaf1 subunits in those two complexes (49). Our study reveals that in the process of incorporating the two functionalities, TIP60 has adopted a previously undescribed arrangement of subunits with respect to its two counterparts, pointing to a distinct mechanism of action on chromatin that may better allow the coordination, efficiency, or regulation of its histone exchange and acetylation activities.

The P400 subcomplex of the TIP60 complex is organized differently from that of the SRCAP complex, which more closely resembles the yeast Swr1 complex. The most notable difference concerns the ARP module, which is intimately associated with the hexameric AAA-ATPase in the P400 subcomplex. This contrasts with its counterpart in the SRCAP complex, where the ARP module is more flexible and not visible in the absence of nucleosomes (65), whereas it becomes visible as it interacts with the nucleosome. Our CX-MS results suggest that, upon binding to the nucleosome, the ARP module of the P400 subcomplex may also undergo large conformational changes to interact with the nucleosome, but the rearrangement seems to be still distinct from that adopted by SRCAP. Further structural and functional studies will be required to elucidate the detailed mechanisms by which the TIP60 complex engages the nucleosome and utilizes ATP hydrolysis to drive the exchange activity of the H2A.Z-H2B dimer.

Differences in architecture with respect to related complexes extend to the incorporation of the transcription factor–interacting subunit, the large TRRAP protein, which, in both the homologous yeast NuA4 and the coactivator SAGA, is rigidly attached to the rest of those complexes. By contrast, a long flexible linker of EP400 connects TRRAP to the rest of TIP60, allowing a far reach of the acetylation and histone exchange functionalities from the site of recruitment. Of notice, the SANT and HD domains, which follow the EP400 linker, occupy the footprint of the structural component SUPT20H on the surface of the TRRAP subunit of the human SAGA complex (69), thereby preventing the formation of a hybrid complex. Our genomic results show that the specific loss of TRRAP in the TIP60 complex leads to a substantial redistribution of H2A.Z and its acetylation with correlating effects on gene expression, both negative and positive. We propose that this is due to defects in the recruitment of TIP60 at specific sites, leading to its action at others.

It has been shown previously that depletion of EP400 by siRNAs leads to large effects on chromatin structure at regulatory elements and both decreased and increased expression of specific genes and noncoding RNA in embryonic stem cells (90, 91). Although it is possible that these large effects are linked to the longer depletion approach, our structure demonstrates the crucial scaffolding role of EP400 within the TIP60 complex, indicating that its depletion should lead to disassembly of the complex in vivo. Therefore, studies performing EP400 knockdowns are most likely measuring the loss of function of the full TIP60 complex instead of its specific role in H2A.Z deposition. The structure of the TIP60 complex presented in this study provides important clues and avenues to more precisely dissect TIP60 function and its molecular mechanisms and distinguish them from other complexes sharing subunits or overlapping roles, such as the SRCAP and SAGA complexes.

TIP60 plays crucial roles in epigenetic mechanisms and cell fate. The structural and functional data reported in this study contribute to the molecular understanding of its assembly and structure and are relevant to a multiplicity of human diseases, including cancer and neurological disorders. Thus, our structural insights hold the potential to help in the development of strategies to regulate TIP60 in different cellular pathways and in contexts of relevance to human pathologies and therapies.

## Materials and methods

### Purification of the different protein complexes used in this study

Engineered human K562 cell lines to purify endogenous TIP60 complexes were described in (28). The EPC1–3Flag-2Strep–expressing cell line was used to purify the native complex and image it to obtain a 3D construction by cryo-EM. Cell lines expressing mutant or truncated subunits of the TIP60 complex from the *AAVS1* safe harbor locus were prepared as described in (28) after mutagenesis in the targeting vector following standard procedures. Affinity purification of native complexes from nuclear extracts was performed as described in (92). The Flag peptide elution step was used for structure, MS, and biochemical analysis. The purified fractions were concentrated 10-fold using Microcon-30 (Millipore) and used to prepare the cryo-EM grids for imaging.

The SRCAP complex was purified from a cell line engineered to express SRCAP-3Flag-2Strep from the *AAVS1* safe harbor locus. Purified fractions were prepared and analyzed at the Proteomics Platform of the Quebec Genomic Center using an Orbitrap Fusion mass spectrometer (Thermo Fisher Scientific) as previously described (30).

For the reconstitution of the P400 subcomplex, RUVBL1, RUVBL2, DMAP1, VPS72, ACTB, BAF53a, EP400, and EPC1 gene fragments were amplified from HeLa cell complementary DNA and inserted into the pCAG vector with either an N-terminal Flag tag or no tag. All the P400 subcomplex vectors were confirmed through sequencing. The plasmids were transfected into Expi293 cells using the Expi293 Expression System Kit (ThermoFisher). The cells were then cultured at 37°C with 8% CO_2_ for 72 hours post transfection. All the reconstituted P400 subcomplexes, including mutants, were purified using the same purification method. First, the cells were harvested and lysed through sonication in a buffer containing 150mM NaCl, 20mM Tris at pH 8.0, 1mM TCEP, 5mM MgCl2, 0.01% NP40, and protease inhibitors. The cell lysate was clarified by centrifugation at 40,000 rpm for 1 hour. Subsequently, the supernatant was collected and incubated with M2 Flag beads (Sigma) for 3 hours. Following incubation, the beads were washed three times with the lysis buffer and eluted with 0.2 mg/ml flag peptide. Later, the protein samples were loaded onto a Q anion-exchange column (Cytiva) equilibrated by a buffer composed of 150 mM NaCl, 20 mM HEPES (pH 7.6), 5 mM MgCl2, and 1 mM TCEP. To separate the protein complexes from nucleic acid impurities, a salt gradient was used, increasing NaCl concentration up to 2 M in 30 min. Afterward, the protein-rich fractions were concentrated and applied to a glycerol gradient ranging from 10 to 30%, buffered with 150 mM NaCl, 20 mM HEPES (pH 7.6), 5 mM MgCl2, 0.1 mM EDTA, and 1 mM TCEP. The gradient centrifugation was performed at 200,000 RCF in an MLS-50 rotor for 10 hours at 4°C. The highly homogenous fractions from the glycerol gradient were combined and concentrated before being flash-frozen in liquid nitrogen for future use.

### Nucleosome reconstitution and histone swapping assay

Histone exchange assays were carried out as previously reported (62), with minor modifications. First, dinucleosomes using the 601 Widom positioning sequence with a 50-bp linker DNA (50N50N6) were reconstituted from biotinylated DNA and recombinant histones as described (93). 10 nM of dinucleosomes immobilized on streptavidin-coated magnetic beads (Dynabeads M-280 Streptavidin, Invitrogen) was combined ~25 nM of purified native complex (TIP60 or SRCAP) and either1 mM ATP or1U apyrase in 70 mM final KCl concentration Exchange Buffer (25 mM HEPES-KOH, pH 7.6, 0.1 mM EDTA, 5 mM MgCl_2_, 10% glycerol, 0.02% NP-40, 1 mM DTT, 0.1 mg/ml BSA, and 70 mM KCl). To overcome sub stoichiometry of dimers associated with the complex, 30 nM of exogenous recombinant H2A.Z.2-H2B dimers was added specifically to the TIP60 reaction samples. In addition, 150 μM of acetyl-CoA was added when indicated. The samples were incubated for 1h at 37C, followed by two washes with 400 mM KCl Exchange Buffer, and two washes with 70 mM KCl Exchange Buffer. Proteins were eluted in SDS–polyacrylamide gel electrophoresis (PAGE) loading buffer then run on 15% gel for Western blot analysis. Antibodies used in this study are listed in table S1.

### HAT assay

For the assay with the native TIP60 complex, 10 nM of the protein was incubated with 100 nM nucleosome in the presence of 50 μM acetyl-CoA within a reaction buffer consisting of 40 mM HEPES pH 7.6, 30 mM NaCl, 0.1 mg/ml BSA, and 10 mM sodium butyrate. The mixture was incubated at 37°C for 2 min, after which it was analyzed by SDS-PAGE gel and immunoblotting. HAT activity was detected with an anti-H4K5ac antibody.

### Chemical cross-linking and MS

Purified human TIP60 complexes (~100 μg) were crosslinked by using 1 mM BS3 (Thermo Scientific) in a buffer consisting of 150 mM NaCl, 25 mM HEPES-KOH (pH 7.6), 0.1 mM EDTA, 5 mM MgCl_2_, 10% glycerol, 0.02% NP-40, and 1 mM DTT for 2 hours at 23°C. Similarly, the P400 subcomplex (~75 μg) was incubated with nucleosomes in a molar ratio of 1:1.5 on ice for 30 min in the presence of 1 mM ATPγS. The reaction was in a buffer containing 70 mM NaCl, 25 mM HEPES-KOH (pH 7.6), 0.1 mM EDTA, 5 mM MgCl_2_, 10% glycerol, 0.02% NP-40, and 1 mM DTT. Subsequently, the mixture was crosslinked with 0.5 mM BS3 for 2 hours at 23°C. Sample processing and MS data analyses were done as previously described (94). The plink2 and Nexus databases were used to search a database composed of the subunit sequences (95, 96).

### Negative staining and cryo-EM sample preparation

In negative stain studies, 3 μl of sample was applied to plasma-cleaned copper grids covered with a continuous layer of carbon film. The sample was incubated on the grid at room temperature for 2 min then stained with three drops of a 2% (w/v) uranyl formate solution (40 μl in total). The stain was blotted off, and the grid was left to dry in a fume hood. Negatively stained grids were loaded onto a Tecnai T12 microscope (FEI) operated at an acceleration voltage of 120 keV. Images were recorded at a magnification of 43,000× with a 4K TVIPS camera.

For cryo-EM visualization, the endogenously purified full TIP60 complex sample (100 nM) was mixed in a 1:1 ratio with crosslinking buffer consisting of 150 mM NaCl, 20 mM HEPES-KOH at pH 7.9,2 mM MgCl2, 1 mM TCEP, 5% Trehalose, 0.01% NP-40, 2 mM ATPγS, and 2% glutaraldehyde. 3 μl of the mixture was immediately applied to a home-made graphene oxide-covered grid (UltrAuFoil R 1.2/1.3, 300 mesh, Gold) (97) and placed in a humidity chamber for 10 min at 4°C. Subsequently, the grid was washed three times with a solution containing 150 mM NaCl, 20 mM HEPES-KOH at pH 7.9, 5 mM MgCl2, 1 mM TCEP, 1 mM ATPγS, 5% Trehalose, and 0.01% NP-40 and then mounted into a Vitrobot Mark IV (Thermo Fisher Scientific). The grid was blotted for 2.5 s at 5N force and immediately frozen by plunging into liquid ethane. Cryo-EM grids of TRRAP-EP400Cter (100nM) and of the reconstituted P400 subcomplex (600 nM) were prepared in a similar manner. The concentration of glutaraldehyde in the crosslinking buffer was halved, and the incubation time for the crosslinking of the reconstituted P400 subcomplex was set to 1 min.

### Cryo-EM data collection

All grids were initially screened using a Talos Arctica electron microscope (Thermo Fisher Scientific). Data collection for the full TIP60 complex was carried out on a Titan Krios G2i (Thermo Scientific) located in the Cal-Cryo facility. Movies were recorded with SerialEM data collection software (98) using a K3 direct electron detector (Gatan) operating in super resolution mode with a pixel size of 0.595 Å. Each movie was acquired with a total dose of ~50 e/Å^2^ and consisted of 50 frames. Two separate datasets were collected for the full TIP60 complex, with the first dataset consisting of 4052 movies, and the second dataset consisting of 10,299 movies. Data for the TRRAP-EP400Cter and the WT P400 subcomplex sample were collected on a G3i electron microscope (Thermo Scientific) using the SerialEM software (98). Movies were recorded on a K3 direct electron detector (Gatan) in super resolution mode, with a pixel size of 0.525 Å. 14,037 and 13,219 movies were collected, respectively, with a total dose of ~50 e/Å2 and 50 frames per movie. Data for the ΔEP400_Loop and ΔVPS72-C terminus mutants were collected on an Arctica electron microscope (Thermo Scientific) using the SerialEM software (98). Movies were recorded on a K3 direct electron detector (Gatan) in super resolution mode, with a pixel size of 0.57 Å. 1868 and 1863movies were collected, respectively, with a total dose of ~50 e/Å2 and 50 frames per movie.

### Cryo-EM data processing

For the full TIP60 complex data sets, the raw movies were subjected to motion correction and CTF estimation using RELION 3.1 software (99–101). Particle picking was performed using crYOLO 1.8.2 (102) and the data was binned by a factor of four. The binned particles underwent 2D classification in RELION to eliminate graphene oxide edges and damaged particles. The precleaned particle sets were unbinned and imported into CryoSPARC 4.4 (103). Heterogeneous refinement in CryoSPARC was employed to identify a self-consistent set, resulting in a density map corresponding to the P400 subcomplex, with some weaker, diffuse density in its periphery. A total of 517,619 particles from both datasets were combined and reimported into RELION (99). After 3D classification without alignment, one major class, comprising 214,481 particles, yielded a structure with an overall resolution of 3.7 Å after consensus refinement. However, while secondary structures were clearly identifiable through the density, the sidechain information was limited, and low-resolution density was observed surrounding the EP400 motor. To improve the resolution of this region, two rounds of focused 3D classification were performed. Eventually, 22,686 particles yielded a structure with a resolution of 5.28 Å (8.6 Å before masking) that allowed better visualization of features surrounding the ATP-dependent motor of EP400. In parallel and in an attempt to improve the resolution for the ARP and BASE modules of theP400 subcomplex, the 214,481 particles were imported into CryoSPARC for further analysis. Soft masks were generated around the ARP and BASE modules (103), which were subsequently used for Local Refinement in CryoSPARC. This process resulted in reconstructions of the ARP module and the BASE module with resolutions of 3.29 Å (7.4 Å before masking) and 3.32 Å (6.6 Å before masking), respectively, which were used for atomic model building.

For the analysis of the TRRAP module, we imported 14,037 movie files into RELION for motion correction and CTF estimation (99–101) and then used the TRRAP protein structure from human SAGA (PDB: 8H7G) as a reference for particle picking (70). After extensive 2D classification and sorting, five classes were chosen as new references for particle picking. We extracted a total of 2,751,524 particles using a box size of 90 pixels, which was binned by 4, yielding a pixel size of 4.2 Å to speed up the subsequent processing. One round of 3D classification with alignment was conducted to clean the particle set, resulting in a subset of 1,099,157 particles. These particles were reextracted with a box size of 180 pixels (binned by 2) before being imported into CryoSPARC for further analysis (103). In CryoSPARC, two rounds of heterogeneous refinement were performed, leading to the selection of 219,180 particles. These particles were then imported back into RELION for further processing. In RELION (99), we reextracted the particles with a box size of 360 pixels and a pixel size of 1.05 Å. The unbinned particles were refined in RELION using a map generated from the previous heterogeneous refinement (99). After refinement, 3D classification without alignment was applied to clean the particle set further, resulting in a final selection of 62,971 particles. After consensus refinement, a soft mask was created and applied to the FAT and PIKK-like domains for one round of 3D classification without alignment. Finally, 23,442 particles were selected and refined, resulting in a 3.40 Å (6.1 Å before masking) map that was used for model building.

The raw movies of the in vitro reconstituted P400 subcomplex were imported into RELION and subjected to Motion Correction and CTF estimation (99–101). After initial processing, LoG picker was used to pick a total of 9,623,505 particles. The particles were binned by 4 and extracted with a box of 90 pixels. Following 2D classification, 5,608,826 particles were selected for further processing. The selected particles were cleaned by 3D classification with alignment. For the initial 3D classification, three classes were chosen to accelerate the processing. Then the best class was selected and reextracted with a 180-pixel box size. Two additional rounds of 3D classification were conducted, each employing seven classes. Subsequently, 185,545 particles were selected and reextracted using a 360-pixel box size with no binning. The reextracted particles were then imported into CryoSPARC for additional data processing (103). After one round of Heterogeneous Refinement, 166,442 particles were selected and subjected to Non-uniform Refinement, yielding a 2.59 Å (3.4 Å before masking) density map that was used for model building. To enhance the resolution of the BAF53a second copy, the imported particles were refined using Non-uniform Refinement in CryoSPARC. A soft mask was applied to the area corresponding to the second copy of BAF53a, followed by focused 3D classification without alignment to sort out potential conformational heterogeneity. Classes with better resolution were selected for another Non-uniform Refinement. Finally, the same soft mask was utilized for one round of Local Refinement to obtain a higher resolution of the second copy of BAF53a. The density obtained with this procedure fits optimally the sequence of BAF53a but not that of ACTB (fig. S8G).

The cryo-EM data for the ΔEP400_Loop and ΔVPS72 C terminus mutants were processed in CryoSPARC using the same workflow. Initially, raw movies were imported and underwent Motion Correction and CTF estimation (99–101). Later, particle selection was performed using the Blob Picker, followed by a single round of 2D classification to generate templates for precise particle picking. The re-picked particles were extracted with a 90-pixel box at a pixel size of 4.56 Å. After 2D classification, 75,954 and 62,192 particles that exhibited clear secondary structural features were chosen from each dataset for reextraction using a 360-pixel box, with the pixel size of 1.14 Å. For each dataset, an Ab-Initio was used to generate three classes as references without introducing model bias. This was followed by Heterogeneous refinement, where particles were classified into three distinct classes, each displaying different structural features.

### Model building and validation

For the TRRAP module, the TRRAP protein structure (PDB: 8H7G) was manually docked into the cryo-EM map using ChimeraX (104). The structures of the SANT and HD domain of EP400 were predicted by AlphaFold2 and manually docked into their corresponding densities (66, 67). The structure was then manually checked in COOT (105).

To build the structure of the ARP module within the P400 subcomplex, we used AlphaFold2 to predict the structure of each subunit (66, 67). Guided by the yeast NuA4 structure (PDB: 7VVZ) (50), the predicted individual structures were subsequently docked into their corresponding density regions. In cases where homology structures were lacking, such as the PDM of EP400, we manually identified the sequence and built the atomic model from features on the high-resolution map using COOT (105). To build the structure of the BASE module, the structure of the AAA-ATPases from the human SRCAP complex (PDB: 6IGM) was manually docked in the density. The structures of the CTD domain of VPS72, Insertion and Motor domains (HD2 part) of EP400, and monomeric BAF53a were predicted using AlphaFold2 (58, 59), then they were docked into the cryo-EM density in ChimeraX (88) and manually adjusted in COOT (89). The structure of the HD1 domain of EP400 was predicted using AlphaFold2 (58, 59), and together with the VPS72-H2AZ-H2B structure (PDB: 5FUG) (63) was docked into the low resolution cryo-EM density. The models were refined using phenix.real_space_refine with secondary structure restraints (106). Refinement and validation statistics are shown in table S2. Figures were generated using ChimeraX (104) and pyMOL (107).

### Degron cell lines

CRISPR/Cas9 was used in K562 cells for homozygous, C-terminal tagging of EP400 with FKBP12^F36V^-2xHA tag to allow for acute knock out using the dTAG system (108). Ouabain co-selection was used to isolate homozygous isogenic clones as previously described (109) (fig. S16A). One clone (C63) was selected for dTAG system validation (fig. S16B), then used to establish complemented cell lines stably expressing either WT or D2361–2530 EP400 from the *AAVS1* safe harbor locus as reported (28). One isogenic clone of each cell line was selected and used in downstream experiments (WT-complemented C1 and Δ2361–2530–complemented C4) (fig. S16C).

### ChIP-seq

Two biological replicates of the two complemented EP400-dTAG K562 cell lines were first treated with 500nM dTAG^V^-1. Following 24 hours of treatment, cells were pelleted to remove the medium and then resuspended in PBS and crosslinked with 1% formaldehyde for 15 min at room temperature. The crosslinking was quenched with 125 mM glycine for 5 min at room temperature. Cells were again pelleted, then washed twice with cold PBS. For chromatin extraction, pellets were first resuspended in Buffer 1 (10 mM Hepes pH 7.5, 10 mM EDTA pH 8, 0.5 mM EGTA, 0.75% Triton X-100), incubated on ice for 10min, pelleted, then resuspended in Buffer 2 (10 mM Hepes pH 7.5, 1 mM EDTA pH 8, 0.5 mM EGTA, 200 mM NaCl), incubated on ice for 10min, pelleted, then resuspended in Lysis Buffer (150 mM NaCl, 25 mM Tris pH 7.5, 1% Triton-X-100, 0.1% SDS, 0.5% Sodium Deoxycholate, 10 mM Sodium butyrate, protease inhibitor cocktail), and incubated for on ice for 30min before sonicating to shear chromatin to around 200bp. After sonication, samples are centrifuged at high speed for 30 min at 4°C in conventional microcentrifuge, and the chromatin-rich supernatant is used for chromatin immunoprecipitation (ChIP). For ChIP, 100ug of chromatin was incubated with either 3ug anti-H2A.Z (Abcam ab4174) or 0.2ug anti-H2A.Zac (Diagenode C15410173) overnight at 4°C, then 25uL Dynabeads Protein A combined with 5uL Dynabeads Protein G was added to each sample and incubated for 4h at 4°C. The beads were washed for 10min at 4°C with Low Salt Buffer (0.1% SDS, 1% triton x-100, 2mM EDTA, 20mM Tris pH8, 150mM NaCl), High Salt Buffer (0.1% SDS, 1% triton x-100, 2mM EDTA, 20mM Tris pH8, 500mM NaCl), LiCl Buffer (1% NP-40, 1% Sodium deoxycholate, 1 mM EDTA, 10 mM Tris pH8, 250 mM LiCl), followed by two quick washes with Tris-EDTA (TE). Chromatin was eluted with 100uL 1% SDS-TE at 1100 rpm with RNase A digestion at 37°C for 30min, followed by Proteinase K digestion at 55°C overnight (10% input was treated the same). DNA was purified using QIAquick PCR purification Kit (Qiagen 28106) according to the manufacturer’s instructions. Libraries were constructed with KAPA Hyper Prep kit (Roche, 07962363001) according to manufacturer’s instructions and samples were sequenced as paired-end 100bp reads on Illumina NovaSeq 6000. For the ChIP-qPCR experiment, three biological replicates of the wild type and mutant cell lines were treated and 500ug of chromatin was used with 5ug of anti-Flag pre-bound to the Protein G Dynabeads, as described (4). qPCR was performed on a Roche LightCycler 480 with validated primers around the transcription start site of the *DACH1* and *CCND1* genes.

### ChIP-Seq analysis

Input and H2A.Z and acetyl-H2A.Z (H2A.Zac) ChIP-seq raw reads were quality-checked with FastQC (v0.11.7) (110) and aligned onto the human genome (hg38 assembly) using Bowtie2 (v2.4.5) (111) with the following options:–local –very-sensitive-local –no-unal –no-mixed –no-discordant –phred33 -I 10 -X 700. Bowtie2 .sam output files were converted to .bam format, sorted and indexed with Samtools (v1.15.1) (112). Coverage bigwig files were generated with deepTools bamCoverage (v3.5.1) (113) with the following options:–normalizeUsing RPKM –extendReads –ignoreDuplicates. Reproducibility between biological replicates was assessed by performing Irreproducible Discovery Rate (IDR) analysis (114): we first ran MACS2 (v2.2.7.1) (115) peak calling algorithm with a liberal p-value cutoff (-p 1e-3). We then sorted peaks by p-value before running IDR (v2.0.3) with the following options:–input-file-type narrowPeak –rank p.value. We filtered IDR output to only select peaks with an IDR score <= 0.05 (transformed IDR value >=540), concatenated the IDR-filtered peak files for WT and either the HSA-6mut or the ΔTRRAP EP400 mutant in a single .bed file by bedops –everything (v2.4.41) (115), and finally sorted and merged the concatenated files with bedtools (v2.30.0) (116). These IDR-filtered and merged .bed files were intersected with the differential peak calling output files (see below) to generate heatmaps, box plots and Venn diagrams. Differential peak calling between WT and either EP400 mutant was performed with MACS2 bdgdiff: we first reran MACS2 (v2.2.7.1) (117) peak calling algorithm with –nomodel and fixed fragment size (–extsize 191). We then used the effective sequencing depth obtained from MACS2 output to run MACS2 bdgdiff (−l 250 −g 150) and identified the coordinates of unchanged, depleted, or enriched ChIP-seq peaks in either EP400 mutant compared to WT EP400. MACS2 bdgdiff output .bed files were intersected with IDR-filtered and merged .bed files by bedops (–element-of 25%) and used to generate genome-wide, TSS-proximal and enhancer-proximal heatmaps of H2A.Z and H2A.Zac ChIP-seq signal with deep-Tools (v3.5.1) (113) computeMatrix and plotHeatmap tools. TSS-proximal H2A.Z and H2A.Zac peaks were defined as peaks overlapping a 2-kb window centered around an annotated gene TSS, identified by bedtools closest (-D ref -t all) using GENECODE v32 annotations. Similarly, enhancer-proximal H2A.Z and H2A.Zac peaks were defined as peaks overlapping a 2-kb window centered around enhancers annotated in K562 cells by the EnhancerAtlas 2.0 project (118), after converting hg19 coordinates to hg38 with the UCSC (119) LiftOver tool. ChIP-seq signal box plots were generated with Python (v3.11.5) (120), using Pandas (v2.0.3), Matplotlib (v3.7.2), Seaborn (0.12.2), SciPy (1.11.1) and NumPy (v1.24.3) libraries, starting from deep-Tools computeMatrix output values, summing H2A.Z/H2A.Zac ChIP-seq signal across each peak coordinate, dividing it by the input signal and plotting the resulting ratios. Overlaps between H2A.Z and H2A.Zac peaks were determined by bedops (–element-of 25%) and used to generate Venn diagrams.

### RNA-seq

Three biological replicates of each of the three complemented EP400-dTAG K562 cell lines were first treated with 500nM dTAGV-1. Following 24 hours of treatment, RNA was extracted using Monarch Total RNA Miniprep Kit (NEB T2010S), then mRNA was enriched with NEBNext^®^ Poly(A) mRNA Magnetic Isolation Module (E7490L), and libraries were constructed with KAPA RNA HyperPrep (Roche, 08098107702), all according to manufacturers’ instructions.

### RNA-seq analysis

RNA sequencing (RNA-seq) raw reads were quality-checked with FastQC (v0.11.7) (110) and aligned onto the human genome (hg38 assembly) using STAR RNA-Seq aligner (v2.7.10b) (121), with the following options:–outSJfilterReads Unique –outFilterMultimapNmax 1 –outFilter IntronMotifs RemoveNoncanonical –outSAM-strandField intronMotif. We used Samtools (v1.15.1) (112) to convert STAR output .sam files into .bam files, and to sort them by name (-n). We then counted how many reads overlapped an annotated gene (GENECODE v32 annotations) using HTSeq (v2.0.2) (122) (htseq-count –stranded=reverse –order=name -f bam –additional-attr=gene_name -m union), and used the output counts files to find DEGs with DESeq2 (123), run with default parameters within the Galaxy platform (124). DEGs were called using and adjusted *P* ≤ 0.01, a fold change ≥2 and ≥5 mean counts. *Z*-score heatmaps of DESeq2 normalized counts were generated with the Galaxy online platform (125). Box plots of DESeq2 normalized counts were generated with with Python (v3.11.5) (120), using Pandas (v2.0.3), Matplotlib (v3.7.2), Seaborn (0.12.2), SciPy (1.11.1) and NumPy (v1.24.3) libraries. Gene transcript levels were visualized on the hg38 genome with the Integrative Genomics Viewer (IGV) (126) using the bigwig output files from deepTools^4^ bamCoverage (–binSize 50 –normalizeUsing BPM) (v3.5.1).

### Statistical analysis

Statistical significance of ChIP-seq and RNA-seq signal distribution (box plots) was evaluated with a Mann-Whitney *U* rank sum test (two-sided).

## Supplementary Material

Supplemental material

## Data Availability

The cryo-EM maps of the ARP module, P400 subcomplex of the endogenous TIP60 complex, TRRAP module, reconstituted P400 subcomplex, and second BAF53a have been deposited in the Electron Microscopy Data Bank (EMDB) with accession codes EMD-45252 EMD-45240, EMD-45176, EMD-45206, and EMD-45180, respectively. The coordinates for the corresponding models have been deposited in the Protein Data Bank (PDB) with accession codes 9C6N, 9C62, 9C47, 9C57, and 9C4B, respectively. MS data of purified complexes has been deposited to the public MassIVE database with accession number MSV000093280. CX-MS data has been deposited to the PRIDE database with accession numbers PXD053207 (endogenous TIP60 complex) and PXD053209 (P400 subcomplex with nucleosome). The ChIP-seq and RNA-seq data generated in this publication are available in the National Center for Biotechnology Information’s Gene Expression Omnibus (GEO) database under the SuperSeries accession number GSE261652.
