# Supplementary material for: Structural insights into the human NuA4/TIP60 acetyltransferase and chromatin remodeling complex
Source: Science. Author manuscript; Available in PMC 2025 Apr 14. (PMC11995519; doi:10.1126/science.adl5816)
Supplement: Supplemental material [file NIHMS2068631-supplement-Supplemental_material.pdf]

Science AAAS

Supplementary Materials for

Structural insights into the human NuA4/TIP60 acetyltransferase and chromatin remodeling complex

Zhenlin Yang *et al*.

Corresponding authors: Jacques Côté, jacques.cote@crchudequebec.ulaval.ca; Eva Nogales, enogales@lbl.gov

Science **385**, eadl5816 (2024)
DOI: 10.1126/science.adl5816

The PDF file includes:

Figs. S1 to S18
Tables S1 to S3

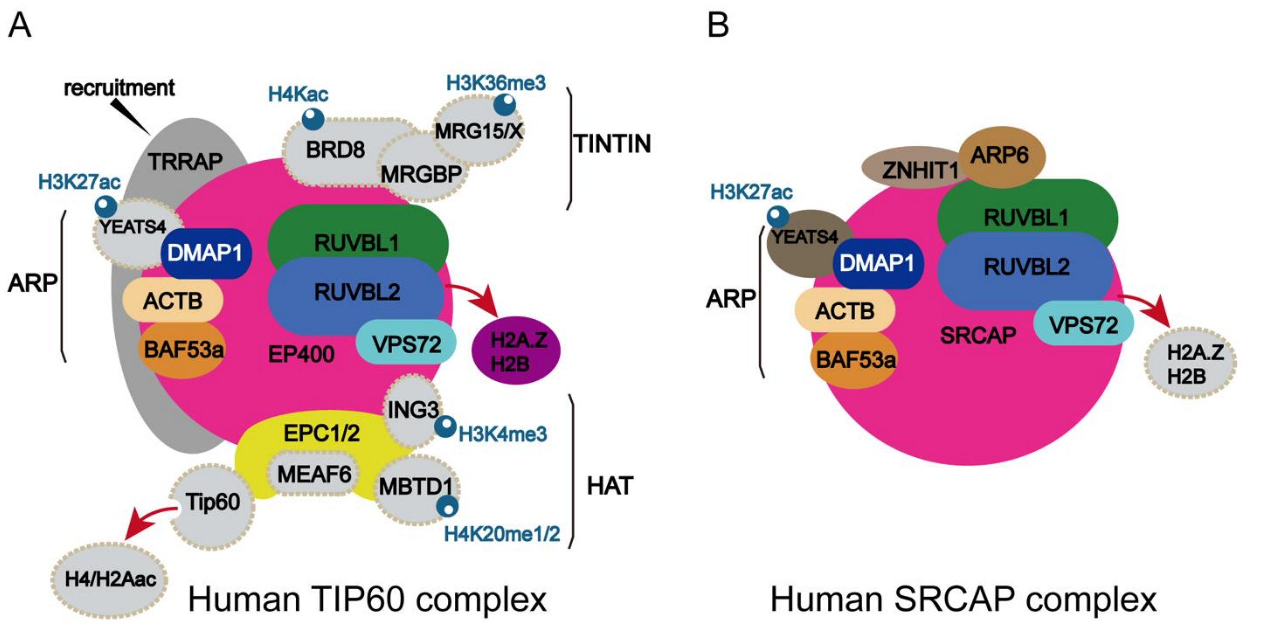

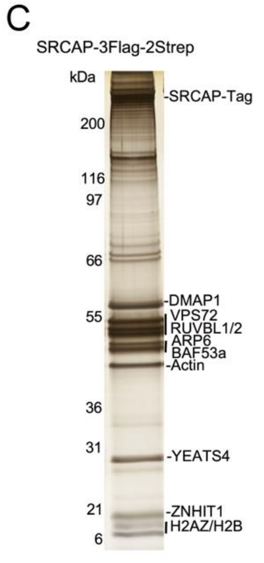

D

subunits	Spectral counts
SRCAP	653
RUVBL2	610
RUVBL1	562
BAF53a	118
DMAP1	116
ARP6	89
ACTB	80
VPS72	73
YEATS4	54
ZNHIT1	34
H2B	33
H2A.Z	16

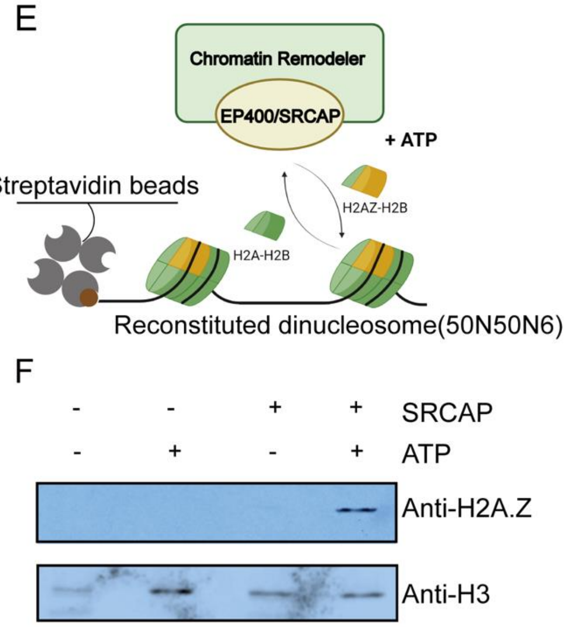

Fig. S1 Subunit composition of functional modules in the TIP60 and SRCAP complexes and biochemical characterization of SRCAP. (A) Schematic view of TIP60 subunits with annotations concerning their functions in histone acetylation, histone exchange, and recognition of multiple histone marks. Subunits labeled by dashed lines (colored in light grey) are not visible in our structure. **(B)** Schematic view of the subunit arrangement in the human SRCAP complex. The H2A.Z/H2B dimer is not present in the reported structure (PDB: 8X19) and is therefore indicated with a dashed line. **(C)** Silver stained SDS-PAGE gel of purified native human SRCAP complex. **(D)** Mass spectrometry results for the sample presented in (C). **(E)** Schematic of our histone exchange assay. **(F)** Immunoblot showing the incorporation of H2A.Z into nucleosomes by SRCAP.

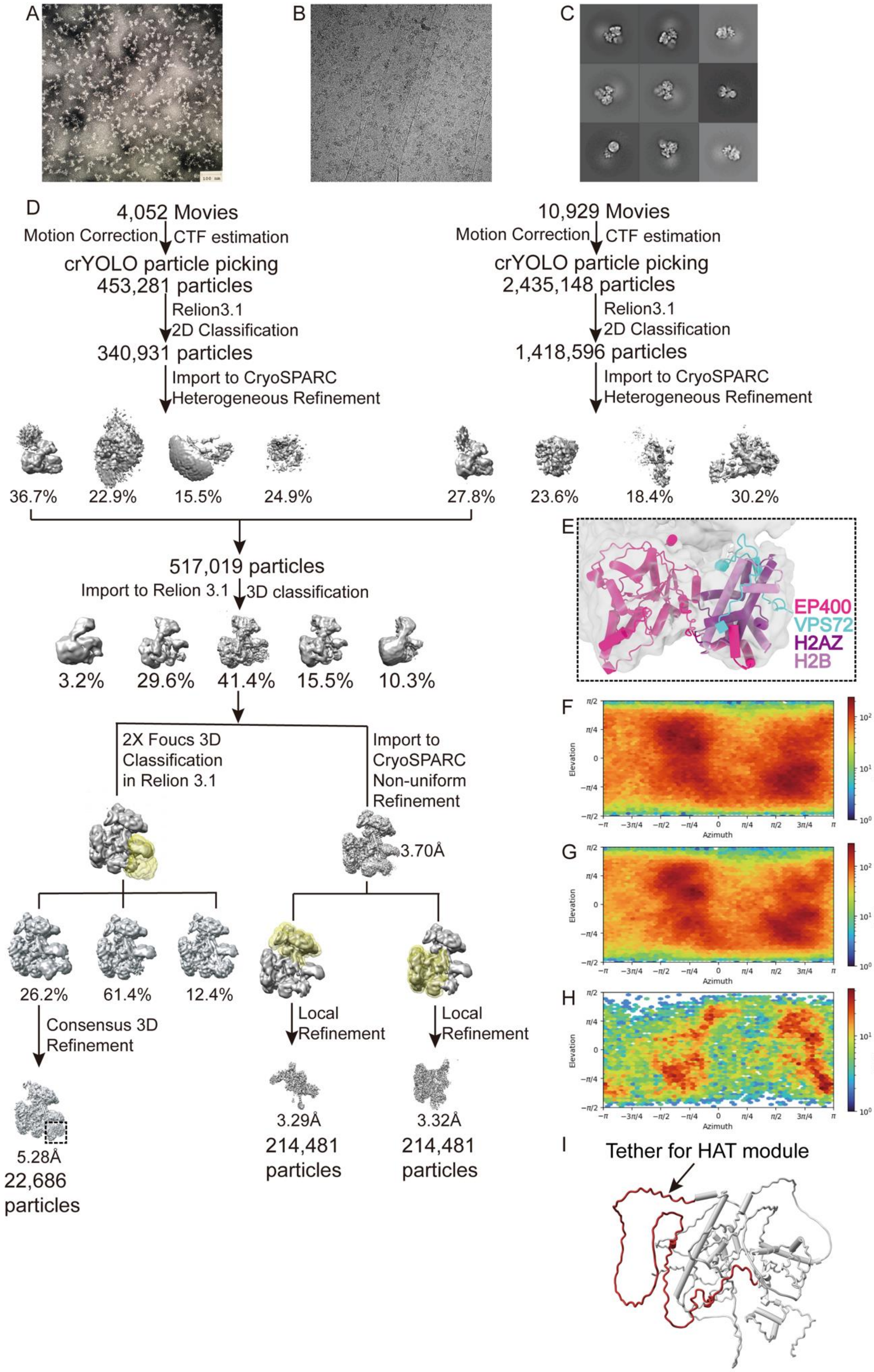
A
B
C
D
4,052 Movies
Motion Correction
CTF estimation
crYOLO particle picking
453,281 particles
Relion3.1
2D Classification
340,931 particles
Import to CryoSPARC
Heterogeneous Refinement
36.7%
22.9%
15.5%
24.9%
10,929 Movies
Motion Correction
CTF estimation
crYOLO particle picking
2,435,148 particles
Relion3.1
2D Classification
1,418,596 particles
Import to CryoSPARC
Heterogeneous Refinement
27.8%
23.6%
18.4%
30.2%
517,019 particles
Import to Relion 3.1
3D classification
3.2%
29.6%
41.4%
15.5%
10.3%
2X Foucs 3D Classification in Relion 3.1
Import to CryoSPARC Non-uniform Refinement
3.70Å
26.2%
61.4%
12.4%
Consensus 3D Refinement
5.28Å
22,686 particles
Local Refinement
3.29Å
214,481 particles
Local Refinement
3.32Å
214,481 particles
E
EP400
VPS72
H2AZ
H2B
F
G
H
Elevation
Azimuth
# of images
I
Tether for HAT module

Fig. S2. Cryo-EM analysis of the endogenous human NuA4/TIP60 complex. (A) Negative stain image of the TIP60 complex. **(B)** Cryo-EM image of the TIP60 complex. **(C)** Representative 2D class averages. The P400 subcomplex can be clearly visualized, while the rest of the complex appears as blurred density due to flexibility. (**D)** Cryo-EM data processing workflow for the TIP60 complex. **(E)** Rigid body fitting was performed on the AlphaFold-predicted multimeric structure using the sequences of H2A.Z, H2B, a fragment of VPS72, and P400, aligning it with the corresponding local density. (**F-H)** Angular distribution of the final 3D reconstructions of the ARP (3.29 Å resolution), BASE (3.32 Å resolution) modules, and the P400 subcomplex after focus classification to select for complexes including the VPS72-H2AZ-H2B (5.28 Å resolution). **(I)** The EPC1 structure is predicted by AlphaFold (the flexible tether for the HAT module is highlighted in red).

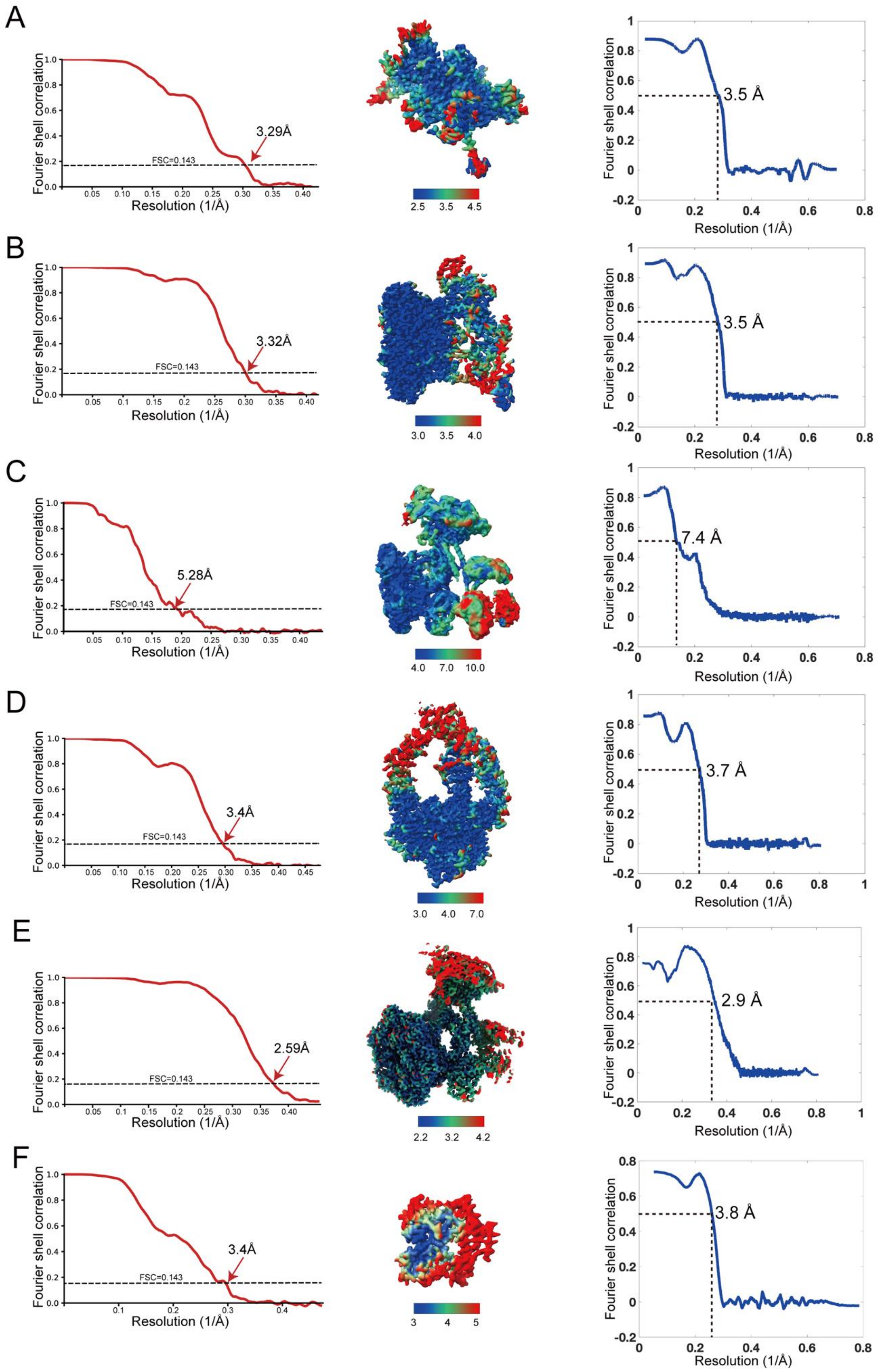
A
B
C
D
E
F
Fourier shell correlation
Resolution (1/Å)
FSC=0.143
3.29Å
3.32Å
5.28Å
3.4Å
2.59Å
3.4Å
3.5 Å
3.5 Å
7.4 Å
3.7 Å
2.9 Å
3.8 Å
2.5 3.5 4.5
3.0 3.5 4.0
4.0 7.0 10.0
3.0 4.0 7.0
2.2 3.2 4.2
3 4 5

Fig. S3. Map quality analysis for the TIP60 modules obtained from different samples and reconstruction schemes through the study. **(A)** ARP module, **(B)** BASE module, **(C)** P400 subcomplex after focus classification to select for complexes including the VPS72-H2A.Z/H2B, **(D)** TRRAP, **(E)** reconstituted P400 subcomplex and **(F)** second copy of BAF53 in the reconstituted P400 subcomplex. Left column shows FSC curves, middle column shows the structures colored by local resolution, and right column shows model vs. map FSC curves using the Phenix real-space refined model.

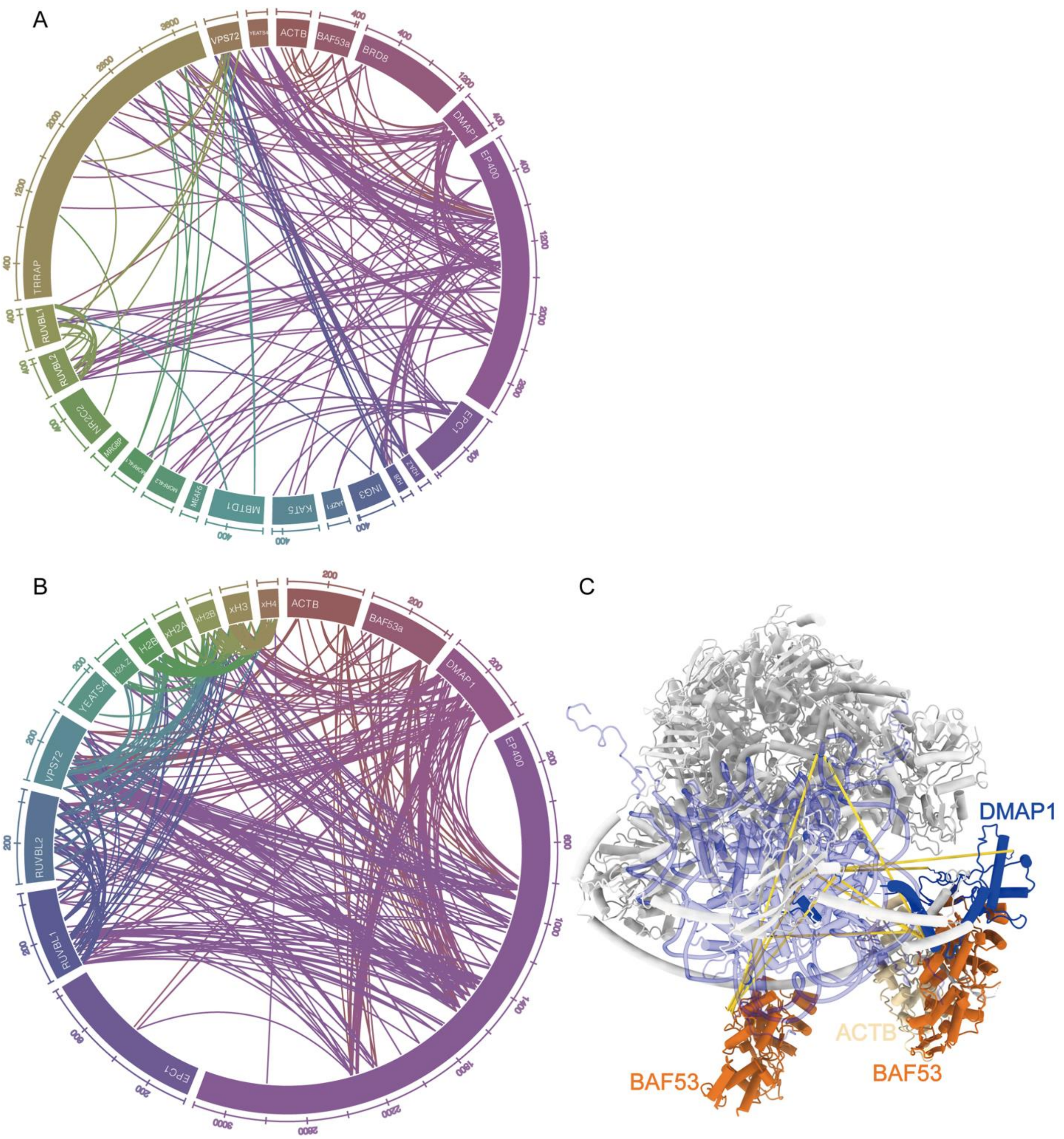

Fig. S4. High-confidence lysine-lysine inter-molecular interactions detected by CX-MS. **(A)** CX-MS results for purified, native TIP60 complexes are illustrated using a circular format. All of the subunits present in Fig. 1B can be localized in the CX-MS result. **(B)** CX-MS results for the reconstituted P400 subcomplex, in the presence of nucleosome and ATPγS. More crosslinks were identified with the P400 complex compared to the native complex, likely due to the higher protein concentration used in the P400 experiment. **(C)** The interactions between DMAP1, ACTB and BAF53a with the nucleosome core, as shown in Panel B, were localized within the SRCAP-NCP structure (PDB: 8X19). Here, BAF53a replaces monomeric actin at the same site, aligning with our TIP60 structure where BAF53a occupies the corresponding region. Ten of the eleven crosslinks (yellow sticks) exceeded a distance of 35 Å, indicating that the crosslinks between the ARP module and the nucleosome depicted in Panel B are not compatible with the SRCAP-NCP structure.

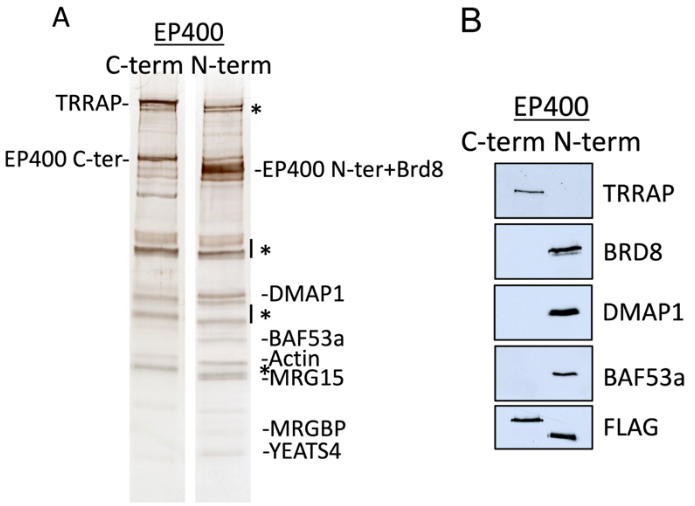

C

Subunits	Spectral counts for EP400 C-term (aa2188-3159)	Spectral counts for EP400 N-term (aa1-964)
TRRAP	1362	
EP400	312	305
ACTB	101	200
RUVBL2	28	50
RUVBL1	20	40
BAF53a	9	148
DMAP1	6	224
BRD8	4	337
MRG15		93
MRGX		68
MRGBP		60
YEATS4		56

Fig. S5. N- and C-terminal portions of EP400 outside of the SWI/SNF ATPase domain assemble partial complexes, neither one of which contains the HAT module. **(A)** Silver-stained gel of purified fractions obtained from K562 cells expressing the C-terminal or N-terminal parts of EP400 from the *AAVS1* safe harbor (aa2188-3159 or aa1-964(isoform 2)). Associated TIP60 subunits identified by mass spectrometry and western blots are labeled on each side. More contaminants (indicated by asterisks) are detected in these FLAG eluates likely because of the expression of artificial truncated proteins (* for HSPs, PRMT5/MEP50 and low amount of RUVBL1/2). **(B)** Immunoblot showing the presence of TRRAP in the sample containing the C-terminal part of EP400, and the TINTIN and ARP modules in the sample containing the N-terminal part of EP400. **(C)** Mass spectrometry analysis of the purified fractions. The peptides corresponding to BAF53a, DMAP1 and BRD8 detected in the C-terminal fraction are most likely due to carry over from the N-terminal fraction. Related to Fig. 1, D and E.

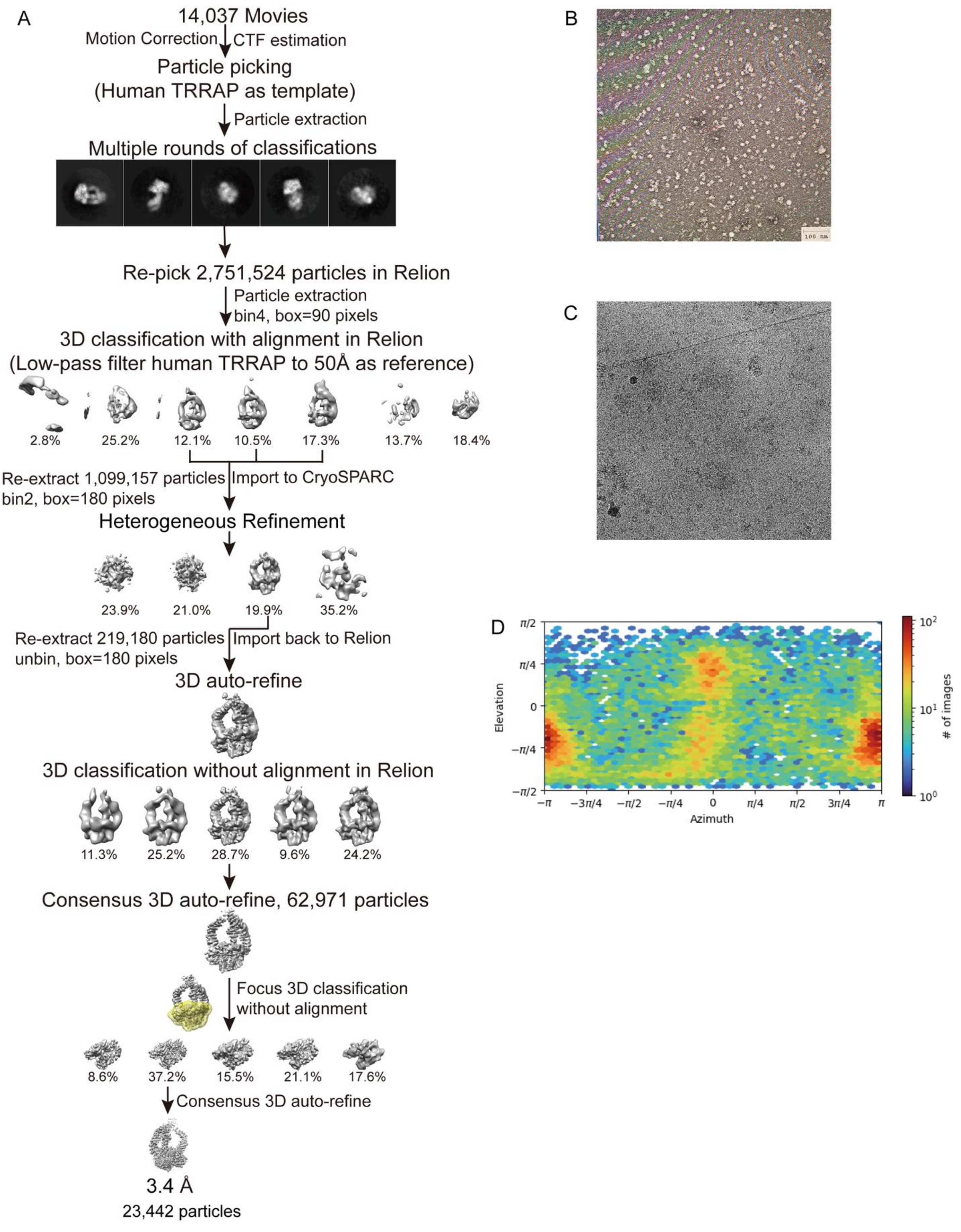

Fig. S6. Cryo-EM analysis of the TRRAP module. (A) Cryo-EM data processing workflow for the TRRAP module. **(B)** Negative stain image of the TRRAP module. **(C)** Cryo-EM image of the TRRAP module. (**D**) Angular distribution of the final reconstruction (overall resolution 3.4 Å).

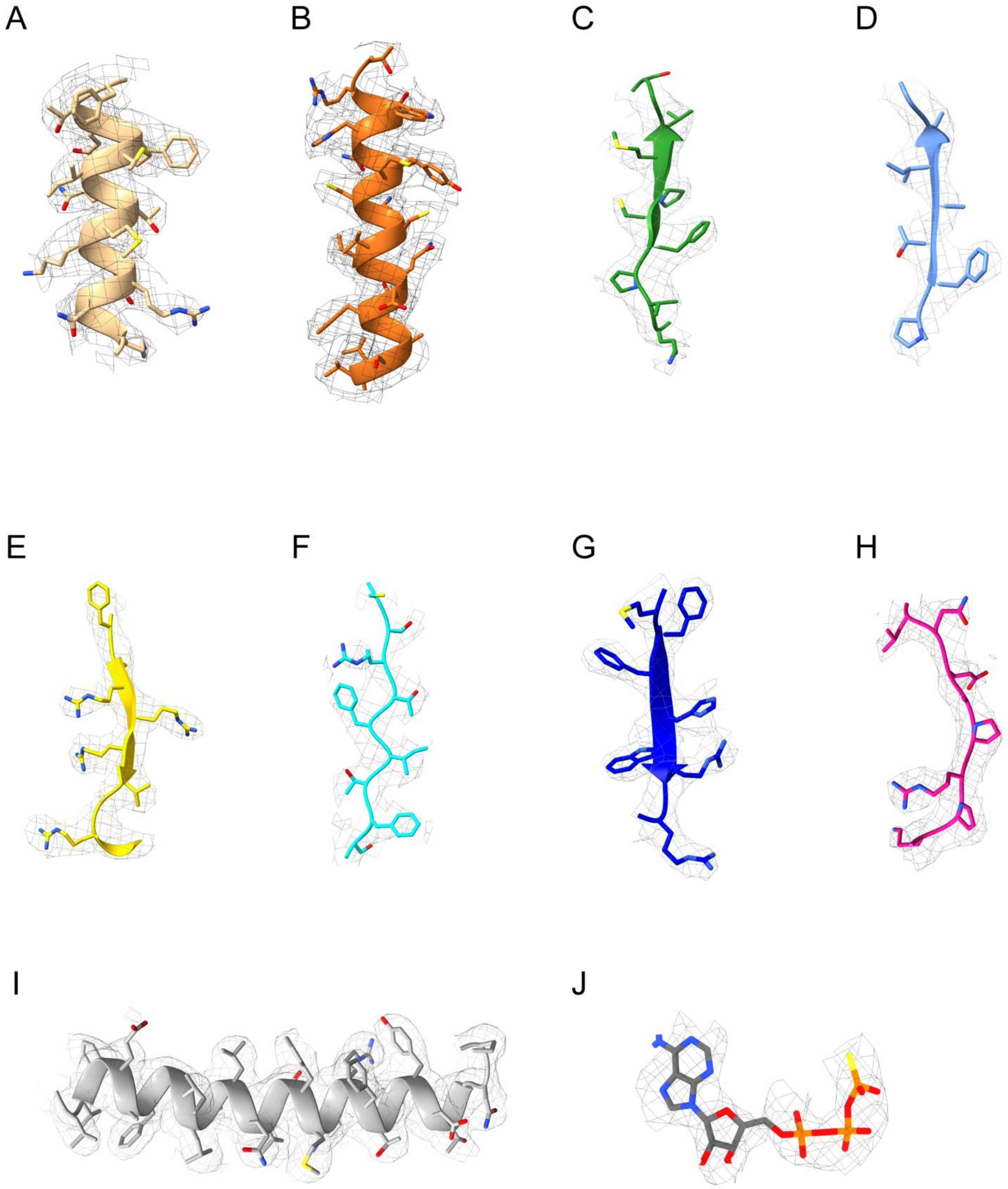

Fig. S7. Representative densities and models for regions in different components of our structures. **(A)** ACTB. **(B)** BAF53a. **(C)** RUVBL1. **(D)** RUVBL2. **(E)** EPC1. **(F)** VPS72. **(G)** DMAP1. **(H)** P400. **(I)** TRRAP. **(J)** ATPγS in the motor domain of the P400 subcomplex.

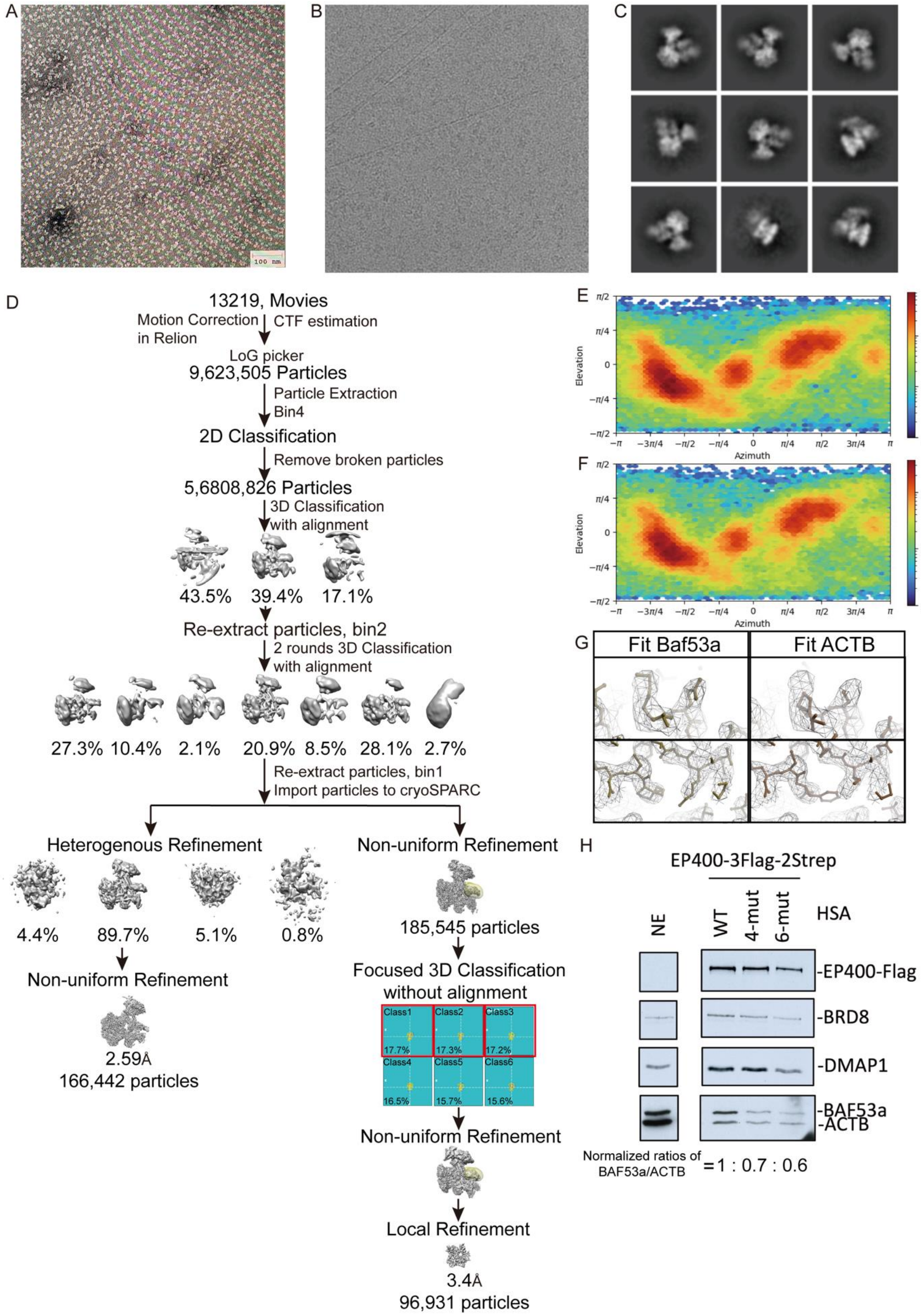

A
100 nm
B
C
D
13219, Movies
Motion Correction in Relion
CTF estimation
LoG picker
9,623,505 Particles
Particle Extraction
Bin4
2D Classification
Remove broken particles
5,6808,826 Particles
3D Classification with alignment
43.5% 39.4% 17.1%
Re-extract particles, bin2
2 rounds 3D Classification with alignment
27.3% 10.4% 2.1% 20.9% 8.5% 28.1% 2.7%
Re-extract particles, bin1
Import particles to cryoSPARC
Heterogenous Refinement
4.4% 89.7% 5.1% 0.8%
Non-uniform Refinement
2.59Å
166,442 particles
Non-uniform Refinement
185,545 particles
Focused 3D Classification without alignment
Class1 17.7%
Class2 17.3%
Class3 17.2%
Class4 16.5%
Class5 15.7%
Class6 15.6%
Non-uniform Refinement
Local Refinement
3.4Å
96,931 particles
E
F
Elevation
Azimuth
# of images
G
Fit Baf53a
Fit ACTB
H
EP400-3Flag-2Strep
NE
WT
4-mut
6-mut
HSA
-EP400-Flag
-BRD8
-DMAP1
-BAF53a
-ACTB
Normalized ratios of BAF53a/ACTB
=1 : 0.7 : 0.6

Fig. S8. Cryo-EM analysis of the reconstituted P400 subcomplex. (A-C) Representative negative stain image, cryo-EM image and 2D class averages of the P400 subcomplex. **(D)** Cryo-EM data processing workflow for the P400 subcomplex. **(E-F)** Angular distribution of the final 3D reconstruction for the P400 subcomplex and the second copy of BAF53a. **(G)** Cryo-EM density map for the assigned second BAF53a copy with atomic models for BAF53a and ACTB showing the optimal fit of the former. **(H)** Western blot analysis of tandem affinity purified fractions from cells expressing EP400 wild type, carrying 4 point mutations (I869D/A870D/A874D/I877D) or carrying 6 point mutations (I869D/A870D/A874D/I877D/F880D/W881D) from the AAVS1 safe harbor. Signal in initial wild type nuclear extract is shown as reference. Comparing BAF53a and actin signals indicate a preferential loss of BAF53a relative to the ratio in the wild type of fraction. Comparative analysis of BAF53a and actin signals indicates a preferential reduction of BAF53a relative to actin in the mutants compared to the wild type, with normalized ratios of BAF53a to ACTB in wild type, 4-mutant, and 6-mutant samples being 1, 0.7, and 0.6, respectively. The ratios of DMAP1 to EP400-flag are consistently 1.9 in the three samples.

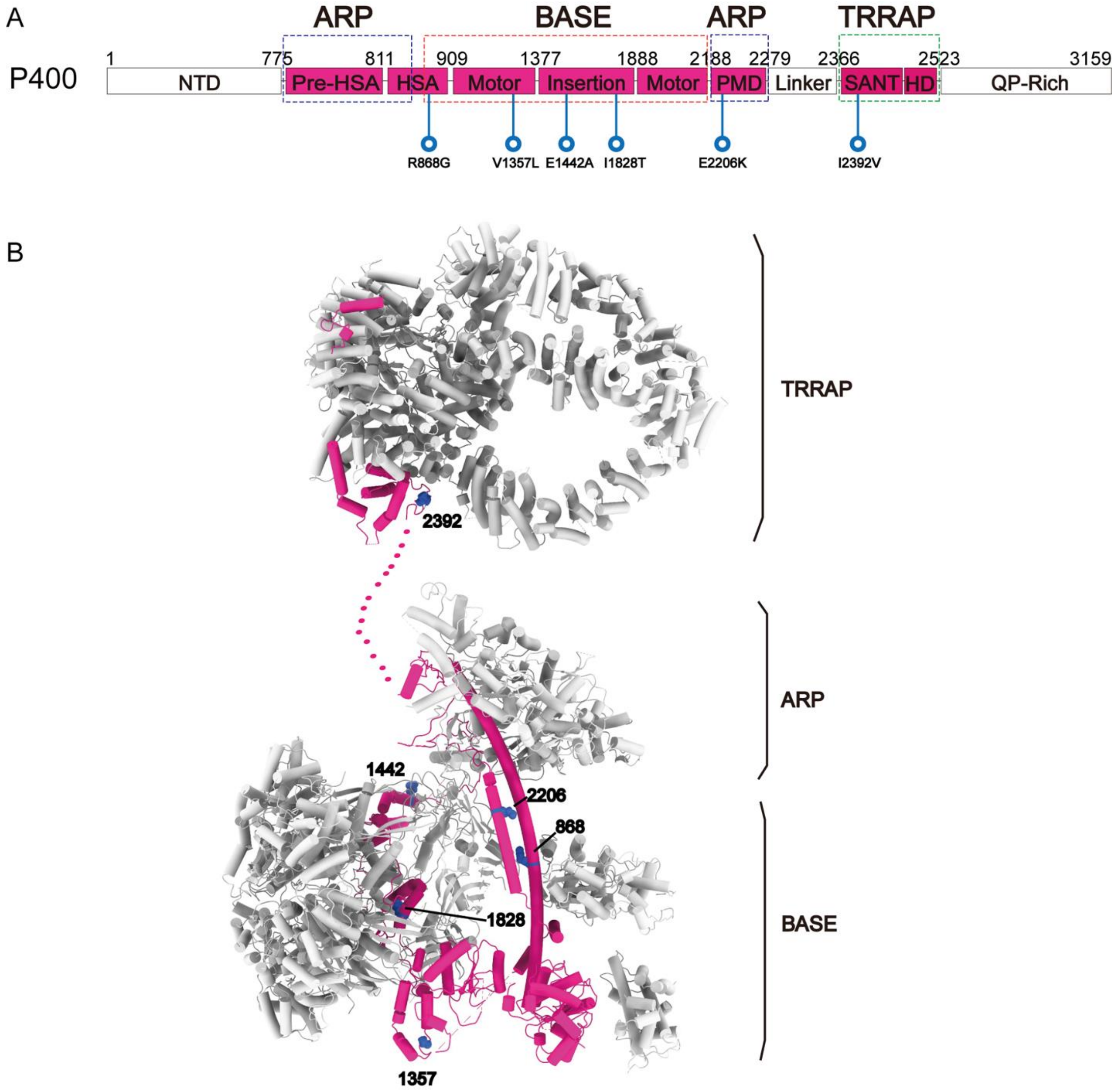

Fig. S9. Localization of EP400 mutations associated with schizophrenia in the Japanese population. (A) Mutations shown on the schematic of EP400 domain structure. **(B)** Localization of the mutations on the 3D structure. EP400 is shown in hot pink, while mutations are shown in blue, and the rest of the complex is shown in grey.

A

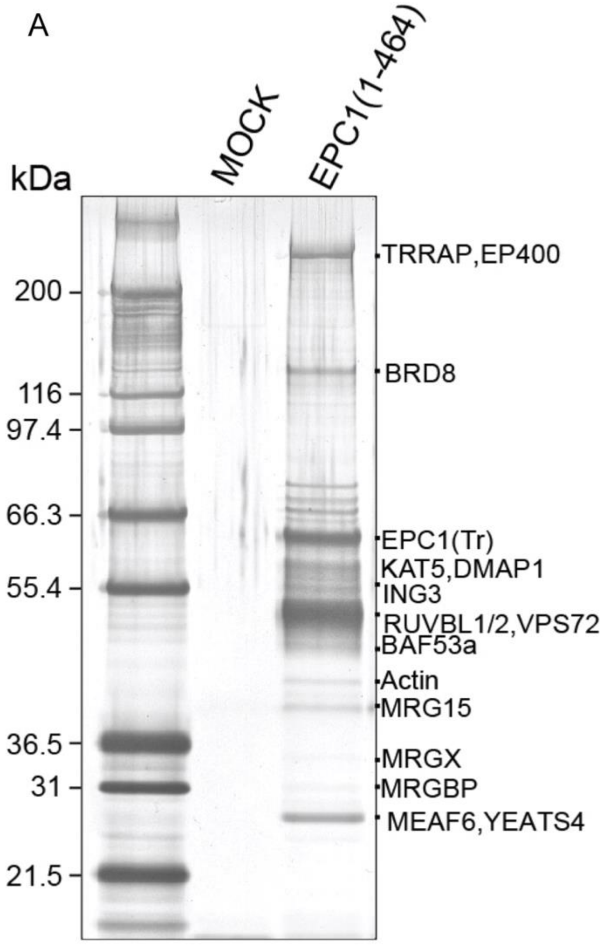

B

subunits	Spectral counts
TRRAP	376
EPC1	286
EP400	240
ING3	213
Tip60	208
RUVBL2	94
RUVBL1	85
BRD8	42
BAF53a	38
DMAP1	31
ACTB	30
MEAF6	28
YEATS4	11
MRG15	11
MRGBP	9
MRGX	5

Fig. S10. The N-terminal residues of EPC1 up to aa464 are sufficient to anchor the HAT module to the rest of the TIP60 complex. (**A**) Silver-stained gel of EPC1(1-464)-containing TIP60 complex tandem affinity purified from K562 cells expressing it from the *AAVS1* safe harbor locus. A mock purification was done in parallel from control untagged cells. Protein bands corresponding to TIP60 subunits are labeled on the right. **(B)** Mass spectrometry analysis of the purified fraction. The only subunit missing known to associate with the EPC1 C-terminal region (*4*, *91*) is MBTD1. Related to Fig. 2, B and F.

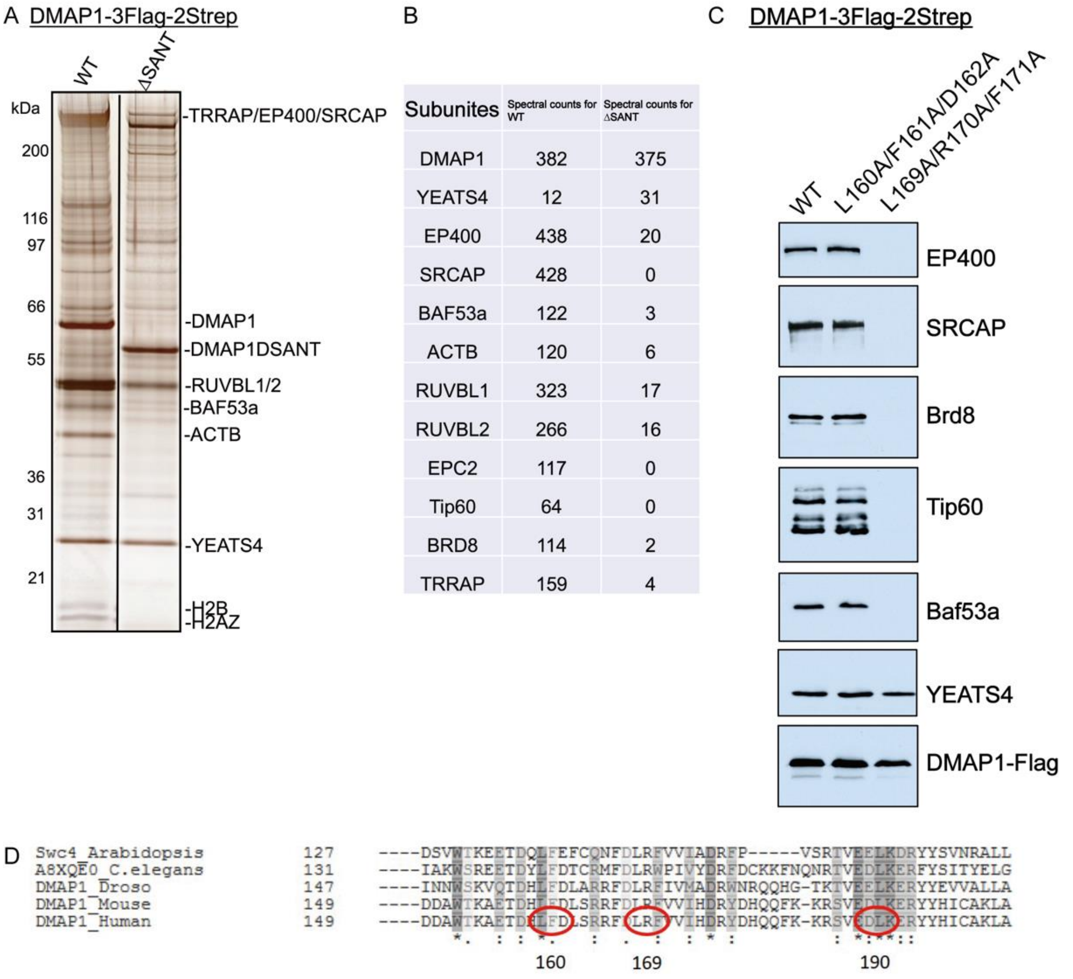

Subunites	Spectral counts for WT	Spectral counts for ΔSANT
DMAP1	382	375
YEATS4	12	31
EP400	438	20
SRCAP	428	0
BAF53a	122	3
ACTB	120	6
RUVBL1	323	17
RUVBL2	266	16
EPC2	117	0
Tip60	64	0
BRD8	114	2
TRRAP	159	4

Fig. S11. The DMAP1 SANT domain is necessary for its association with the TIP60 complex (and SRCAP) while it retains its association with YEATS4/GAS41. (A) Silver-stained gel of tandem affinity purified DMAP1 wild type or DMAP1 ΔSANT (aa149-199) from K562 cells expressing them from the *AAVS1* safe harbor locus. Protein bands corresponding to TIP60 subunits (and SRCAP) are labelled on the right. **(B)** Mass spectrometry analysis of the purified fractions. Related to Fig. 3G. **(C)** Western blot analysis of tandem affinity purified fractions from cells expressing DMAP1 wild type, carrying mutations of L160A/F161A/D162A and L169A/R170A/F171A from the *AAVS1* safe harbor. **(D)** Sequence alignment of the SANT domain of DMAP1 across different species. Mutations of the conserved amino acids are labeled with red ovals.

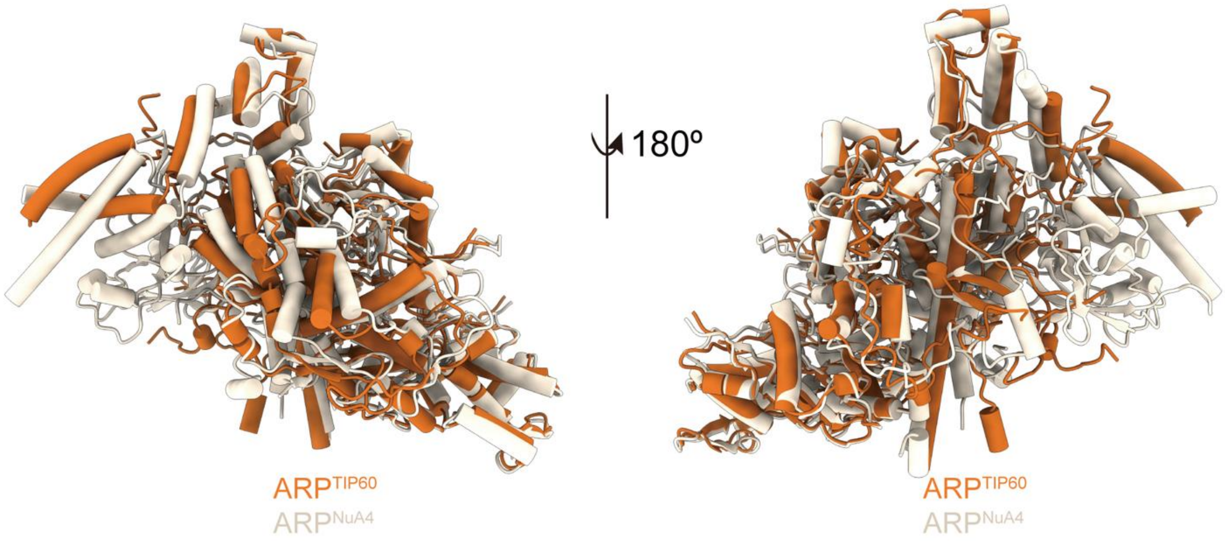

Fig. S12. Structural comparison of the ARP module of human TIP60 and yeast NuA4. Superposition of the ARP modules of TIP60 (brown) and NuA4 (beige). RMDS = 1.1 Å.

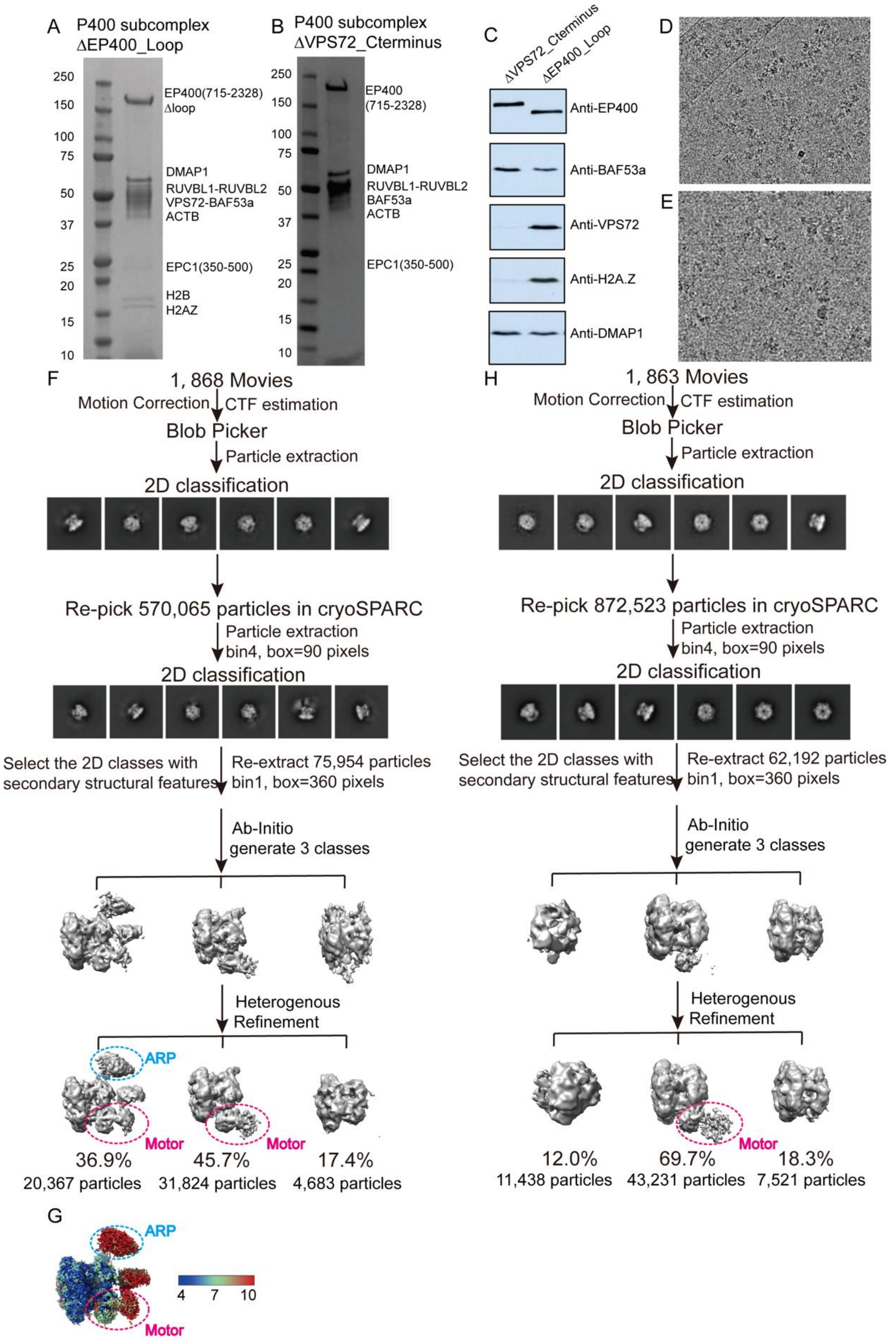

A
P400 subcomplex
ΔEP400_Loop
250
150
100
75
50
37
25
20
15
10
EP400(715-2328)
Δloop
DMAP1
RUVBL1-RUVBL2
VPS72-BAF53a
ACTB
EPC1(350-500)
H2B
H2AZ
B
P400 subcomplex
ΔVPS72_Cterminus
EP400
(715-2328)
DMAP1
RUVBL1-RUVBL2
BAF53a
ACTB
EPC1(350-500)
C
ΔVPS72_Cterminus
ΔEP400_Loop
Anti-EP400
Anti-BAF53a
Anti-VPS72
Anti-H2A.Z
Anti-DMAP1
D
E
F
1, 868 Movies
Motion Correction
CTF estimation
Blob Picker
Particle extraction
2D classification
Re-pick 570,065 particles in cryoSPARC
Particle extraction
bin4, box=90 pixels
2D classification
Select the 2D classes with
secondary structural features
Re-extract 75,954 particles
bin1, box=360 pixels
Ab-Initio
generate 3 classes
Heterogenous
Refinement
ARP
Motor
Motor
36.9%
20,367 particles
45.7%
31,824 particles
17.4%
4,683 particles
G
ARP
Motor
4
7
10
H
1, 863 Movies
Motion Correction
CTF estimation
Blob Picker
Particle extraction
2D classification
Re-pick 872,523 particles in cryoSPARC
Particle extraction
bin4, box=90 pixels
2D classification
Select the 2D classes with
secondary structural features
Re-extract 62,192 particles
bin1, box=360 pixels
Ab-Initio
generate 3 classes
Heterogenous
Refinement
Motor
12.0%
11,438 particles
69.7%
43,231 particles
18.3%
7,521 particles

Fig. S13. Cryo-EM analysis of reconstituted P400 subcomplex mutants. (A, B) SDS-PAGE gel depicting the purified ΔEP400_Loop and ΔVPS72 C-terminus mutants, respectively. **(C)** Western blot validation of the mutants shown in panels A and B indicates that DMAP1 and BAF53 are present in both mutants, while VPS72 and H2A.Z were depleted in the ΔVPS72 C-terminus mutant. **(D, E)** Representative Cryo-EM micrographs of the ΔEP400_Loop and ΔVPS72 C-terminus mutants. **(F, H)** Cryo-EM data processing workflows for the ΔEP400_Loop and ΔVPS72 C-terminus mutants, respectively. **(G)** Local resolution estimation for the initial class of ΔEP400_Loop mutant reconstruction, indicating a significantly reduced resolution in the ARP module as compared to the rest of the complex, suggesting increased flexibility of the ARP module due to the EP400_Loop deletion.

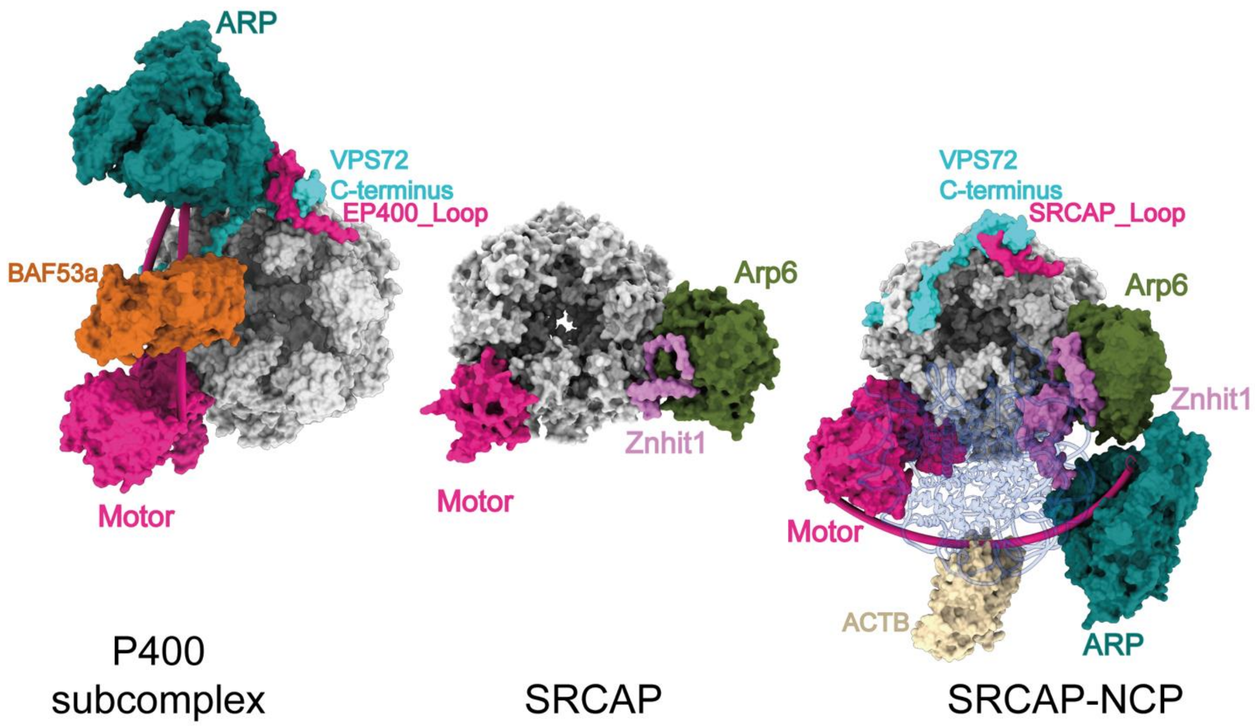

Fig. S14. Side-by-side structural comparison between the P400 subcomplex described in this work (left), SRCAP complex (middle, PDB: 6IGM) and SRCAP-NCP complex (right, PDB: 8X19). The motors (hot pink) are positioned so as to be approximately aligned to each other. Only the ARP module (turquoise), Arp6 (army green), AAA-ATPase hexamer (grey), motor and HAS domains of the remodeler (hot pink), BAF53a (brunt orange), VPS72 C-terminus (cyan), ZNHIT1 (orchid) and ACTB (wheat) are shown for clarity. The nucleosome engaged by SRCAP is shown for reference in grey ribbon. The VPS72 C-terminus, the SRCAP-Loop and the ARP module are not visible in the nucleosome-free structure of SRCAP.

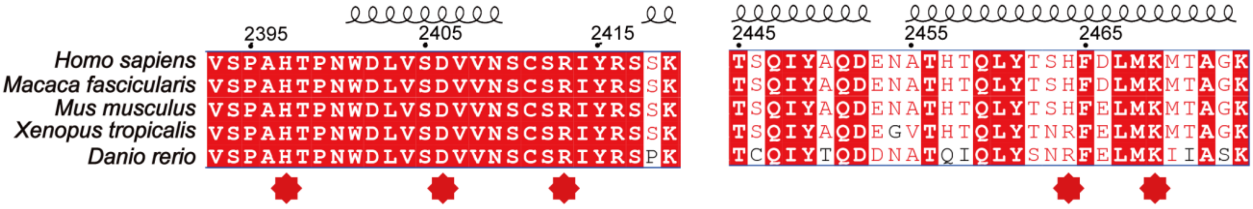

Fig. S15. Conservation analysis of the SANT domain of EP400. Amino acids shown in (Fig.4C) are marked with octagons.

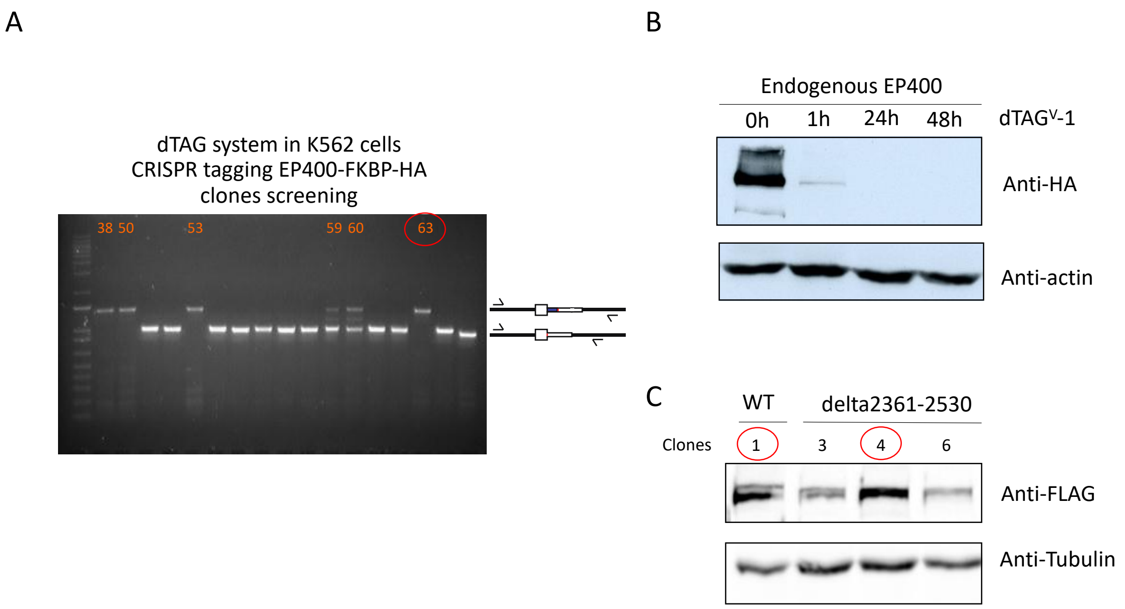

Fig. S16. CRISPR-mediated construction of an endogenous EP400 degron cell line and its complementation with WT and mutant EP400 expressed for the AAVS1 locus. (A) Agarose gel of out-out PCR on clones after CRISPR/Cas9 using gRNAs targeting the C-terminus of EP400 to introduce the FKBP12F36V-2HA cassette. Heterozygote and homozygote edited clones were obtained and homozygote clone 63 was selected for characterization. **(B)** Anti-HA western blot to show rapid acute depletion of endogenous EP400 after adding dTAG-V1 to the media. **(C)** Anti-Flag western of clones expressing WT and mutant EP400 from the AAVS1 locus (after genome editing of clone 63) to select clones with similar levels of expression (circled).

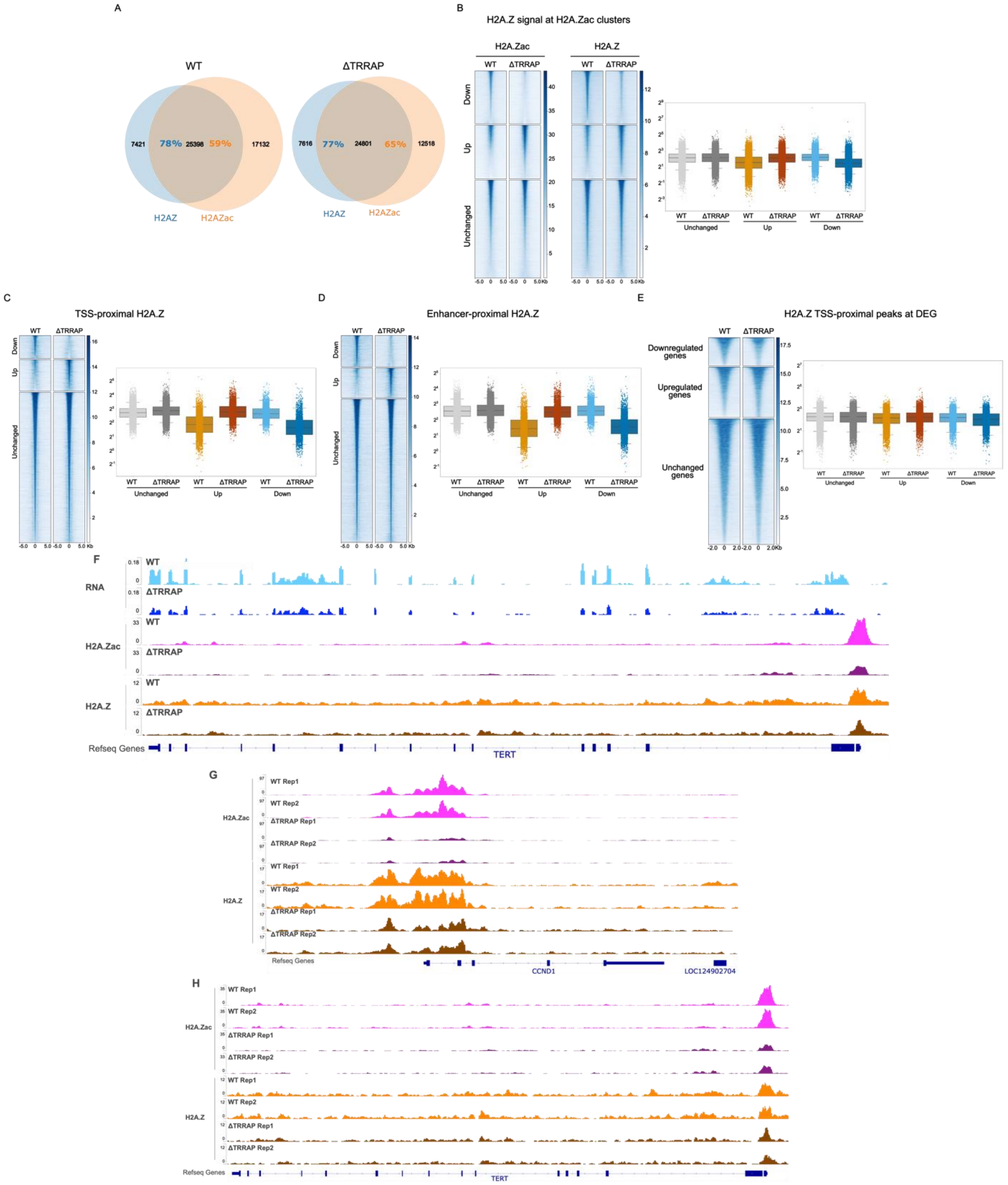
A
WT
ΔTRRAP
7421
78%
25398
59%
17132
7616
77%
24801
65%
12518
H2AZ
H2AZac
B
H2A.Z signal at H2A.Zac clusters
H2A.Zac
H2A.Z
WT
ΔTRRAP
Down
Up
Unchanged
C
TSS-proximal H2A.Z
D
Enhancer-proximal H2A.Z
E
H2A.Z TSS-proximal peaks at DEG
Downregulated genes
Upregulated genes
Unchanged genes
F
RNA
H2A.Zac
H2A.Z
Refseq Genes
TERT
G
WT Rep1
WT Rep2
ΔTRRAP Rep1
ΔTRRAP Rep2
CCND1
LOC124902704
H

Fig. S17. Coordinated changes in H2A.Z and H2A.Zac genome-wide occupancy in response to TRRAP dissociation. (A) Venn diagrams showing overlaps between H2A.Z and H2A.Zac peaks in both the WT (left) and the ΔTRRAP mutant (right) conditions. **(B)** Heatmap and box plot representation of H2A.Z peaks that overlap H2A.Zac peak clusters for ΔTRRAP mutant vs WT. **(C)** TSS-proximal H2A.Z peaks (TSS +/- 2Kb) clustered according to change in the mutant vs WT, represented by a heatmap (left) showing signal +/- 5Kb from peak center, and a box plot (right). **(D)** Similar representation of enhancer-proximal H2A.Z peaks (enhancer +/- 5Kb). **(E)** Heatmap and box plot representation of H2A.Z ChIP-seq signal for peaks that are located near the TSS (+/- 2Kb) of DEGs. **(F)** Genome browser view of normalized read counts of RNA-seq, H2A.Zac ChIP-seq and H2A.Z ChIP-seq over the gene *TERT.* **(G)** Genome browser view of normalized read counts of H2A.Zac and H2A.Z ChIP-seq biological replicates over the gene CCND1. **(H)** Genome browser view of normalized read counts of H2A.Zac and H2A.Z ChIP-seq biological replicates over the gene TERT**.** Two-sided Mann Whitney U test was performed on all the box plots, and the p values are indicated in Table S3.

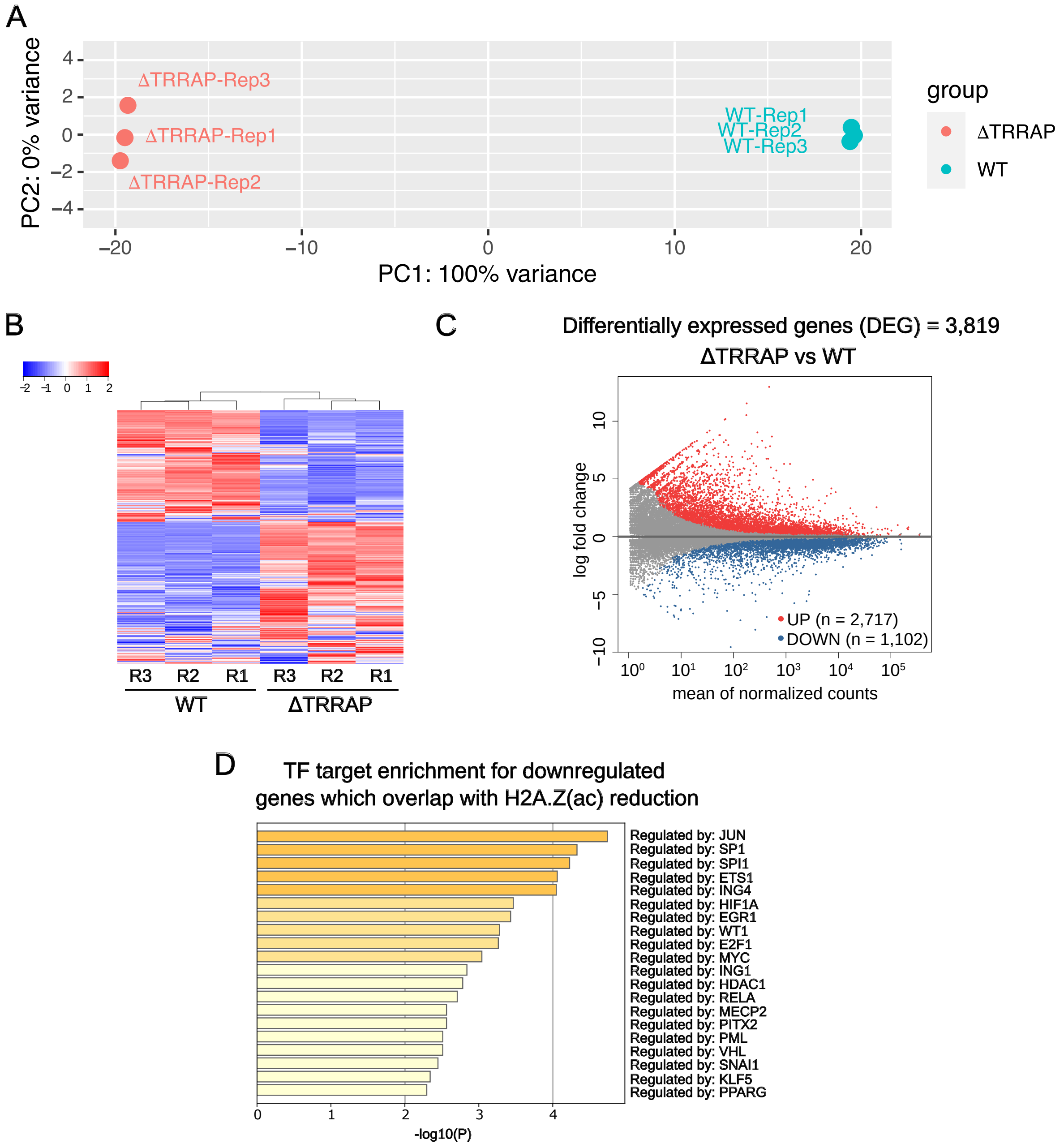

Fig. S18. RNA-seq: ΔTRRAP vs WT. (A) PCA analysis for biological replicates of the three conditions (n=3 each). Virtually all of the sample variability is described by the 1st principal component. **(B)** Z-score heatmaps of normalized transcript counts for ΔTRRAP vs WT**. (C)** MA plots showing significantly upregulated (red) or downregulated (blue) genes, with normalized counts for each. **(D)** Metascape-generated TRRUST analysis on the downregulated genes that show reduced H2A.Z(ac) signal in the ΔTRRAP mutant vs WT. The “Down” gene category identified in Fig. 5D shows enrichment of genes regulated by specific transcription factors.

Table S1 Antibodies used in this study.

FLAG M2-Peroxidase	Sigma-Aldrich, Cat # A8592, RRID:AB_439702
EP400	Abcam, Cat # ab5201, RRID:AB_304780
Tip60	Santa Cruz Biotechnology, Cat # sc-5727, RRID:AB_2128817
DMAP1	Thermo Fisher Scientific, Cat # PA1-886, RRID:AB_2292767
YEATS4	Santa Cruz Biotechnology, Cat# sc-393708
BAF53a	Abcam, Cat # ab3882, RRID:AB_304125
ActinB	Bioshop, Cat # TLC002.100
BRD8	Bethyl Laboratories, Cat # A300-219A, RRID:AB_263340
Histone H2B	Abcam, Cat # ab1790, RRID:AB_302612
Histone H2A.Z	Abcam, Cat # ab4174, RRID:AB_304345
Histone H3	Abcam, Cat # ab1791, RRID:AB_302613
Histone H4K5ac	Thermo Fisher Scientific, Cat # PA1-84334, RRID: AB_931272
TRRAP	Santa Cruz Biotechnology, Cat# sc-5405
HA	Roche, Cat # 12013819001
Tublin	Millipore, Cat # CP06
Actin	Fisher, Cat #AM4302
SRCAP	Gift of J. Chrivia

Table S2 Cryo-EM data collection, processing, and refinement.

	ARP module (PDB: 9C6N) (EMDB: 45252)	P400 Subcomlex (PDB: 9C62) (EMDB: 45240)	TRRAP module (PDB: 9C47) (EMDB: 45176)	Recombinant P400 Subcomplex (PDB: 9C57) (EMDB: 45206)	Second BAF53a (PDB: 9C4B) (EMDB: 45180)
**Data collection and processing**					
Microscope	Krios	Krios	Krios	Krios	Krios
Voltage (keV)	300	300	300	300	300
Camera	K3	K3	K3	K3	K3
Magnification	64,000	64,000	81,000	81,000	81,000
Pixel size at detector (Å/pixel)	1.19	1.19	1.05	1.05	1.05
Total electron exposure (e^{-}/Å^2)	50	50	50	50	50
Number of frames/exposure	50	50	50	50	50
Defocus range (μm)	-1 ~ -2	-1 ~ -2	-1 ~ -2	-1 ~ -2	-1 ~ -2
Automation software	SerialEM	SerialEM	SerialEM	SerialEM	SerialEM
Micrographs collected (no.)	14,351	14,351	14,037	13,219	13,219
Micrographs used (no.)	14,351	14,351	14,037	13,219	13,219
Total extracted particles (no.)	2,888,429	2,888,429	2,751,524	9,623,505	9,623,505
**For each reconstruction:**					
Refined particles (no.)	214,481	214,481	214,481	214,481	214,481
Final particles (no.)	214,481	214,481	214,481	214,481	214,481
Point-group	C1	C1	C1	C1	C1
Resolution (global, Å)					
FSC 0.5 (unmasked/masked)	7.4/3.3	8.6/5.3	6.1/3.7	3.4/2.7	5.9/3.4
FSC 0.143 (unmasked/masked)	7.4/3.29	8.6/5.28	6.1/3.4	3.4/2.59	5.9/3.4
Resolution range (local, Å)	2.5 - 4.5	4.0 - 10.0	3.0 - 7.0	2.2 - 4.2	3.0 - 5.0
Map sharpening *B* factor (Å^2)	77	207	84	77.7	83.8
Map sharpening methods	cryoSPARC	cryoSPARC	cryoSPARC	cryoSPARC	cryoSPARC
**Model Composition**					
Protein	5	15	2	15	1
Ligand	2	8	0	8	0
RNA/DNA	0	0	0	0	0
**Model Refinement**					
Refinement package	Phenix	Phenix	Phenix	Phenix	Phenix
real or reciprocal space	Real	Real	Real	Real	Real
resolution cutoff (Å)	3.3	5.3	3.4	2.6	3.4
Model-Map scores					
CC	0.85	0.77	0.78	0.84	0.84
Average FSC (Å)	3.5	7.4	3.7	2.9	3.9
B factor (Å^2)					
Protein residues	83.6	339	111.9	25.84	97
Ligands	99.31	158.98	---	75.91	---
R.m.s. deviations from ideal values					
Bond length (Å)	0.008	0.01	0.002	0.003	0.003
Bond angles (º)	0.609	0.58	0.518	0.658	0.551
**Validation**					
MolProbity score	2.25	2.26	1.94	2.28	1.96
CaBLAM outliers	2.57	3.15	0.21	2.89	3.15
Clash score	22.18	21.3	10.17	10.55	9.84
Poor rotamers (%)	0	3.46	0.28	2.56	0.3

Ramachandran plot					
Favored (%)	93.8	93.3	93.7	93.32	93.02
Outliers (%)	0.3	0.3	0.3	0.29	0

Table S3 P-values were calculated using the Mann-Whitney U test in this study.

Figure	WT Unchaged vs Mut Unchanged	WT Upregulated vs Mut Upregulated	WT Downregulated vs Mut Downregulated	WT Unchaged vs WT Upregulated	WT Unchanged vs WT Downregulated	WT Upregulated vs WT Downregulated
H2AZ at DEG delta-TRRAP vs WT	4.906e-01	9.731e-14	1.354e-23	2.387e-40	2.259e-11	1.136e-04
H2AZ at H2AZac delta-TRRAP vs WT	2.089e-14	0.000e+00	0.000e+00	0.000e+00	2.276e-40	0.000e+00
H2AZ enhancer-proximal delta-TRRAP vs WT	2.534e-19	0.000e+00	0.000e+00	0.000e+00	8.748e-04	0.000e+00
H2AZ TSS-proximal delta-TRRAP vs WT	2.154e-168	0.000e+00	0.000e+00	0.000e+00	3.103e-07	0.000e+00
H2AZ genome-wide delta-TRRAP vs WT	3.704e-12	0.000e+00	0.000e+00	0.000e+00	2.805e-04	0.000e+00
H2AZac at DEG delta-TRRAP vs WT	2.117e-03	1.974e-62	2.994e-77	0.000e+00	4.719e-44	2.603e-87
H2AZac at H2AZ delta-TRRAP vs WT	9.009e-01	0.000e+00	0.000e+00	0.000e+00	0.000e+00	1.726e-271
H2AZac enhancer-proximal delta-TRRAP vs WT	6.288e-17	0.000e+00	0.000e+00	0.000e+00	5.220e-03	0.000e+00
H2AZac TSS-proximal delta-TRRAP vs WT	4.891e-43	0.000e+00	0.000e+00	8.033e-99	6.826e-117	4.571e-01
H2AZac genome-wide delta-TRRAP vs WT	1.110e-09	0.000e+00	0.000e+00	0.000e+00	3.267e-58	0.000e+00
